# Creatine-mediated ferroptosis inhibition is involved in the intestinal radioprotection of daytime-restricted feeding

**DOI:** 10.1080/19490976.2025.2489072

**Published:** 2025-04-09

**Authors:** Yingjuan He, Gaomei Zhao, Xue Ouyang, Shaobo Wang, Yin Chen, Chenwenya Li, Yongwu He, Jining Gao, Songling Han, Jinghong Zhao, Junping Wang, Cheng Wang

**Affiliations:** a State Key Laboratory of Trauma and Chemical Poisoning, Institute of Combined Injury of PLA, College of Preventive Medicine, Army Medical University, Chongqing, China; b Department of Nephrology, Xinqiao Hospital, Army Medical University, Chongqing, China

**Keywords:** Ionizing radiation-induced intestinal injury, gut microbiota, ferroptosis, creatine, time-restricted feeding

## Abstract

Ionizing radiation-induced intestinal injury (IRIII) is a catastrophic disease lack of sufficient medical countermeasures currently. Regulation of the gut microbiota through dietary adjustments is a potential strategy to mitigate IRIII. Time-restricted feeding (TRF) is an emerging behavioral nutrition intervention with pleiotropic health benefits. Whether this dietary pattern influences the pathogenesis of IRIII remains vague. We evaluated the impact of TRF on intestinal radiosensitivity in this study and discovered that only daytime TRF (DTRF), not nighttime TRF, could ameliorate intestinal damage in mice that received a high dose of IR. Faecal metagenomic and metabolomic studies revealed that the intestinal creatine level was increased by approximate 9 times by DTRF, to which the *Bifidobacterium pseudolongum* enrichment contribute. Further investigations showed that creatine could activate the energy sensor AMP-activated protein kinase in irradiated enterocytes and induce phosphorylation of acetyl-CoA carboxylase, resulting in reduced production of polyunsaturated fatty acids and reduced ferroptosis after IR. The administration of creatine mitigated IRIII and reduced bacteremia and proinflammatory responses. Blockade of creatine import compromised the ferroptosis inhibition and mitigation of DTRF on IRIII. Our study demonstrates a radioprotective dietary mode that can reshape the gut microbiota and increase intestinal creatine, which can suppress IR-induced ferroptosis, thereby providing effective countermeasures for IRIII prevention.

## Introduction

Since the discovery of X-rays by Roentgen in 1895, nuclear energy and technology have been widely applied in industry, health care and agriculture. In addition to the benefits of ionizing radiation (IR), its potential threat to the public has also been increasingly recognized, particularly after the occurrence of two level 7 (major accident) nuclear power plant accidents, Chernobyl and Fukushima, which caused hundreds of thousands of people to be exposed to excess IR.^1^ The mammalian intestine is one of the most sensitive tissues to IR. If given a high dose of IR, people may suffer from lethal intestinal injury that primarily presents as the loss of intestinal barrier integrity and severe endogenous infections.^2,3^ IR induced intestinal injury (IRIII) is also a common complication in patients with abdominal and pelvic malignant tumors after radiotherapy,^4^ which seriously affects patient’s quality of life. Currently, no drugs are approved by the FDA to treat IRIII, which necessitates the development of effective countermeasures.

The human intestine is colonized by 100 trillion microorganisms that are in a dynamic equilibrium state under physiological conditions.^5^ Advances in microbiome research have revealed that gut microbiota dysbiosis plays a crucial role in the pathogenesis of IRIII.^6,7^ In patients with IR-induced enteritis, gut microbial diversity is reduced, and the observed microbiome disturbances are closely related to symptoms such as diarrhea, fatigue, and systemic inflammatory responses.^8^ Similar results have been reported in mice with IRIII.^3^ Compared with wild-type mice, germ-free mice are more resistant to IRIII.^9^ Additionally, oral supplementation of high-abundance enterobacteria, *Lachnospiraceae* and *Enterococcaceae*, from mice that survive IRIII induces radioprotection,^7^ which indicates the occurrence of microbial regulation of intestinal radiosensitivity.

Alterations in dietary habits affect the composition of the gut microbiota.^10^ Time-restricted feeding (TRF) is an intermittent fasting dietary pattern that limits food consumption to a specific time window (usually 8 ~ 12 h) rather than requiring fasting days. Owing to unlimited energy intake during eating periods and pleiotropic health benefits, including improvements in sleep, glucoregulation, blood pressure, liver triglycerides, plasma lipids, and cardiac function,^11–14^ TRF has received substantial attention in recent years. Mechanistically, an increasing number of studies have indicated that gut microbiota remodeling contributes to the benefits of TRF.^15,16^ This dietary pattern has been discovered to increase the gut microbiota diversity^17^ and the abundance of some radioprotective bacteria, such as *Lactobacillus* and *Bifidobacterium*.^18^ Recently, the improvements in the microbial diversity and the abundance of these beneficial commensal bacteria are revealed instrumental for IRIII treatment,^19^ raising a possibility that TRF may influence intestinal radiosensitivity. Notably, applying TRF during the daytime (the rest period in mice; DTRF) or the nighttime (NTRF) influences the potency of the induced effects on longevity and running endurance.^20,21^ Whether DTRF and NTRF have different impacts on the pathogenesis of IRIII also warrants investigation.

In addition to the gut microbiota dysbiosis, ferroptosis in enterocytes is also an important cause driving the occurrence of IRIII. Ferroptosis is a type of programmed cell death caused by excessive production of lipid peroxides in an Fe-dependent manner.^22^ Mounting evidence suggests that ferroptosis initiation contributes to IR-induced cytotoxicity.^23–25^ Accordingly, increased ferroptotic flux sensitizes cancer cells to radiotherapy,^25^ whereas inhibiting IR-induced ferroptosis confers radioprotection.^24^ Application of the ferroptosis inhibitor, Ferrostatin-1 (Fer-1), has been revealed to mitigate the injury of enterocytes caused by IR *in vitro* and *in vivo*.^26,27^ The gut microbiota has regulatory effects on ferroptosis through its microbial composition and metabolites.^28^ Although gut microbiota regulation has been found to mitigate IRIII,^3,6,7^ whether this radioprotective effect correlates to ferroptosis inhibition is unclear.

Limiting food consumption to an 8-h eating window is a highly concerned TRF dietary pattern in clinical practice and has been confirmed can induce weight loss in obese patients^29^ and elicit multiple anti-aging effects.^30^ Focusing on the “diet – gut microbiota – host triangle”,^31^ our study starts with an evaluation of the impact of this TRF habit on intestinal radiosensitivity in mice, followed by a multiomics analysis to probe the underlying microbial and metabolic factors involved. We found that DTRF enabled to reshape the gut microbiota and increase intestinal creatine (Cr) levels. Cr, an arginine (Arg) metabolite that could be upregulated by *Bifidobacterium pseudolongum* (*B. pseudolongum*) in the gut, was identified as a ferroptosis inhibitor by reducing the production of polyunsaturated fatty acids (PUFAs) after IR. Our findings demonstrate that Cr-mediated ferroptosis inhibition contributes to the intestinal radioprotection of DTRF and provide promising solutions for IRIII prevention.

## Results

### DTRF ameliorates the intestinal injury caused by IR

**Figure 1.**
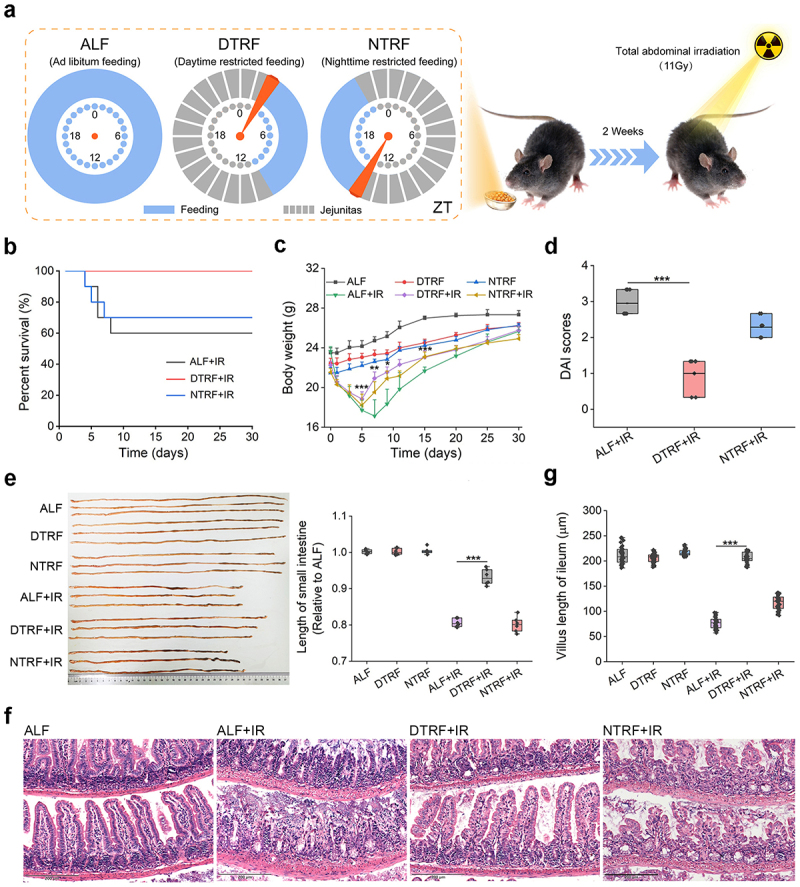
DTRF mitigates IR-induced intestinal injury. (a) Schematic representation of the dietary patterns for radiosensitivity evaluation. The feeding times for DTRF and NTRF are ZT2–10 and ZT14–22, respectively. ALF mice have continuous access to a standard diet. No restriction on the supply of drinking water. (b) Percent survival of mice after TAI. Mouse survival was monitored for 30 days after irradiation. Each group contains 10 mice (*n* = 10). (c) Alterations in mice body weight after TAI. The body weight was determined on days 1, 3, 5, 7, 9, 11, 15, 20, 25 and 30 after irradiation. Results are shown as means + standard deviations (SDs; *n* = 6). *, *p* < 0.05; **, *p* < 0.01; ***, *p* < 0.001, DTRF+IR relative to IR. (d) DAI scores of mice on day 5 after TAI. Results are shown in the boxplot format (*n* = 7). ***, *p* < 0.001. (e) Representative photograph showing changes in the length of small intestine in mice. Relative length of the small intestine is shown in the boxplot (*n* = 6). ***, *p* < 0.001. (f) HE staining revealing the mitigation of DTRF on IRIII in mice. Scale indicates 200 μm. (g) Villous length in mice ileum. Fifty ileal villi were measured for each group. Results are shown in the boxplot. ***, *p* < 0.001.

To investigate the influence of TRF on intestinal radiosensitivity, male mice subjected to DTRF (zeitgeber time [ZT] 2–10), NTRF (ZT14–22), and ad libitum feeding (ALF) for 1, 2 or 3 weeks were evaluated using a total abdominal irradiation (TAI) model induced by 11 Gy γ-ray at ZT1 (Figure 1a), when male mice have been proven to be sensitive to IR.^3^ Despite different time windows for feeding, these three groups of mice consumed similar amounts of chow (4% fat) every day before irradiation (Figure S1a, Supporting Information). In line with the ability of TRF to prevent weight gain in mice fed a normal chow or high-fat diet,^32,33^ DTRF and NTRF both reduced the rise of mice weight compared with ALF (Figure S1b, Supporting Information). However, these two dietary patterns had different impacts on intestinal radiosensitivity. NTRF did not significantly improve the mice survival throughout the experiment (Figure S2, Supporting Information). In contrast, DTRF treatment for 1 week increased the survival of irradiated mice from 60% to 80%, and the mice treated with DTRF for 2 or 3 weeks were all alive (Figure 1b). According to this result, we employed a 2-week TRF treatment for further investigation.

Detection of the body weight indicated that DTRF outperformed NTRF in reducing the weight loss caused by TAI (Figure 1c). Consistent with the findings in irradiated mice,^3,34^ we observed obvious intestinal symptoms such as diarrhea and bloody stools in mice subjected to ALF on Day 5 after TAI. Nevertheless, these symptoms were notably mitigated by DTRF, rather than NTRF, as indicated by a decreased disease activity index (DAI) score (Figure 1d). Measurement of small intestine length revealed evident tissue shrinkage in mice subjected to ALF after TAI (Figure 1e), which corresponded with ileal histopathological alterations, including villous shedding (Figures 1f and S3, Supporting Information), shortened villous length (Figure 1g) and reduced crypt counts (Figure S4, Supporting Information). NTRF failed to alleviate tissue shrinkage and diarrhea symptoms, whereas DTRF promoted radioresistance and improved all pathological changes caused by IR. These findings indicate that only DTRF, not NTRF, can mitigate IRIII.

### B. pseudolongum *enrichment contributes to the radioprotection of DTRF*

**Figure 2.**
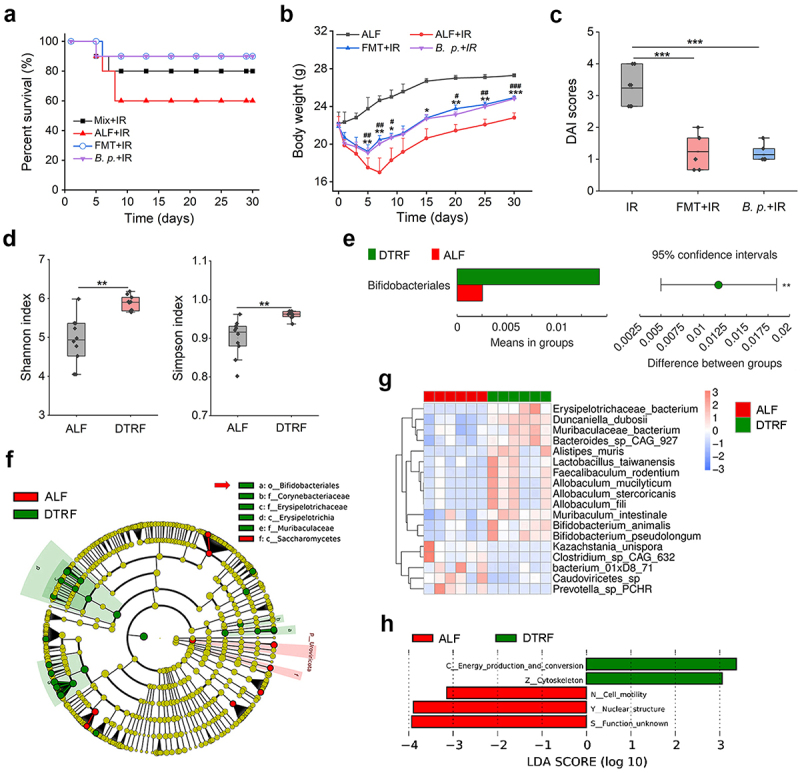
DTRF reshapes the gut microbiota and enriches the radioprotective *B. pseudolongum*. (a) Percent survival of mice after TAI. Mix indicates the group where ALF mice live in the cages with the faeces of DTRF mice. *B.P*., *B. pseudolongum*. Each group contains 10 mice. (b) Alterations in mice weight. Results are shown as means + SDs (*n* = 6). *, *p* < 0.05; **, *p* < 0.01; ***, *p* < 0.001, FMT+IR relative to ALF+IR. ^#^, *p* < 0.05; ^##^, *p* < 0.01; ^###^, *p* < 0.001, *B.P*.+IR relative to ALF+IR. (c) DAI scores of mice on day 5 after TAI. Results are shown in the boxplot (*n* = 7). ***, *p* < 0.001. (d) Shannon and Simpson indices indicating the α diversity of mouse microbiota. Results are shown in the boxplot (*n* = 10). **, *p* < 0.01. (e) t-test analysis showing the effect of DTRF on mouse microbiota at the order level based on the 16S rRNA sequencing data (*n* = 10). **, *p* < 0.01. (f) Cladogram depicting the enrichment of *Bifidobacteriales* by DTRF based on the metagenomic sequencing data (*n* = 6). *Bifidobacteriales* is indicated with a red arrow. (g) LDA heatmap displaying the differential bacteria between the mice subjected to ALF and DTRF. Only the species with LDA scores greater than 4 are presented. (h) Enriched eggNOG terms in the mice subjected to ALF and DTRF.

To gain insight into the radioprotective effect of DTRF, we next performed a dirty-cage experiment and observed that the survival of mice subjected to ALF living in cages with faeces from mice subjected to DTRF after TAI was improved from 60% to 80% (Figure 2a). Additionally, faecal microbiota transplantation (FMT) to transfer the microbiota from mice subjected to DTRF to mice subjected to ALF increased mouse survival from 60% to 90%, along with reduced weight loss (Figure 2b) and intestinal symptoms after TAI (Figure 2c), which suggests that remodeling of the gut microbiota contributes to reduced intestinal toxicity. 16S rRNA sequencing was then employed to analyze the impact of DTRF on the gut microbiota using mice faeces obtained on Day 14 (ZT1) after dietary intervention. The values of two α diversity indices, the Shannon and Simpson indices (Figure 2d), were significantly greater in mice subjected to DTRF than in those subjected to ALF. Analysis of similarities (ANOSIM) revealed a *p* value of 0.0049 (R = 0.74), which indicated greater intergroup differences than within-group differences. Permutational multivariate analysis of variance (PERMANOVA) was also performed and confirmed the difference in microbial β-diversity (weighted UniFrac metrics) between the two groups (pseudo-F = 5.46, *p* = 0.003). The species annotation revealed that DTRF increased the abundance of *Bacteroidota* from 37.87% to 55.37% but decreased the *Firmicutes* abundance from 47.1% to 29.52% (Table S1, Supporting Information). A t test revealed that *Bifidobacteriales* was the only bacteria whose abundance changed (upregulated by DTRF) significantly with a *p* value lower than 0.01 at the order level (*p*  = 0.002, Figure 2e). Improvements in microbial α diversity, the *Bacteroidota*/*Firmicutes* ratio, and the abundance of *Bifidobacterium* were also detected in mice subjected to DTRF compared with mice subjected to ALF after TAI (Figure S5, Supporting Information).

Furthermore, a metagenomic study and the nonparametric test ANOSIM were carried out and revealed the difference in microbial composition at the species level between the two groups (*R* = 0.407, *p* = 0.004). We next conducted a linear discriminant analysis (LDA) effect size (LEfSe, *p* < 0.05, LDA > 4) analysis, which is an instrumental tool for identifying differential intestinal bacteria caused by changes in dietary patterns,^35^ and found the differential abundance of *Bifidobacteriales* between mice subjected to ALF and DTRF (Figure 2f). Specifically, DTRF increased the contents of *B. pseudolongum* and *Bifidobacterium animalis* (*B. animalis*) at the species level (Figure 2g). Bacteria such as *Duncaniella dubosii*, *Bacteroides* sp. *CAG 927*, *Muribaculum intestinale*, and *Alistipes muris* also increased in abundance after DTRF. Notably, *B. pseudolongum*, a probiotic that could promote radioresistance in mice,^36^ was one of the bacteria with the most significantly altered abundance in mice subjected to DTRF compared with mice subjected to ALF (*p*  = 0.0039; Table S2, Supporting Information). Our results validated that oral administration of *B. pseudolongum* improved the survival of irradiated mice from 60% to 90% (Figure 2a) and markedly relieved the weight loss and intestinal symptoms caused by TAI (Figures 2b,c). It is plausible that the increased abundance of *B. pseudolongum* contributes to the radioprotective effect of DTRF.

### DTRF induces an increase in Cr content in the gut

**Figure 3.**
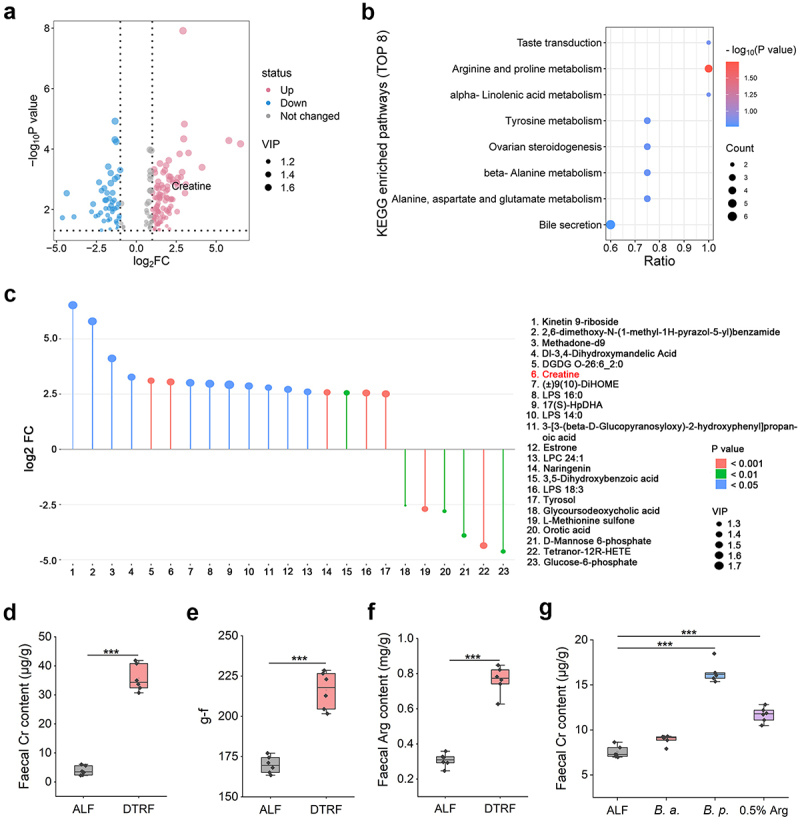
DTRF increases the Cr content in the gut. (a) Volcano plot displaying the differential metabolites in the faeces of mice subjected to ALF and DTRF based on the metabolomic data. (b) Top 8 pathways enriched by KEGG. (c) Stem plot indicating the differential metabolites. Only the metabolites with log2 FC values greater than 2.5 are presented. Cr is listed the sixth metabolite and colored red. (d) Faecal Cr contents in the mice subjected to ALF and DTRF. Results are shown in the boxplot (*n* = 6). ***, *p* < 0.001. (e) Determination of the mice grip strength. Results are shown in the boxplot (*n* = 6). ***, *p* < 0.001. (f) Faecal Arg contents in the mice subjected to ALF and DTRF. Results are shown in the boxplot (*n* = 6). ***, *p* < 0.001. (g) Faecal Cr contents in the mice subjected to ALF after oral administration of *B. animalis*, *B. pseudolongum* and 0.5% Arg. Results are shown in the boxplot (*n* = 6). ***, *p* < 0.001.

Changes in microbiome composition influence the production of gut microbiota metabolites that can affect host physiology. To identify the differentially abundant metabolites in the gut of mice subjected to DTRF, we next conducted a metabolomics study. A total of 169 metabolites (117 with increased abundance, 52 with decreased abundance; Figure S6, Supporting Information) exhibited significant alterations (FC > 2 or FC < 0.5, and *p*  < 0.05, VIP > 1.0) in the faeces of mice subjected to DTRF compared with those of mice subjected to ALF (Figure 3a), as detected in negative ion mode. Arg and proline metabolism was determined to be the sole signaling pathway significantly (*p*  = 0.018) enriched *via* Kyoto Encyclopedia of Genes and Genomes (KEGG) analysis (Figure 3b). Moreover, no significant change was observed for the pathways enriched in the results obtained in positive ion mode (Table S3, Supporting Information). Metabolomic profiling revealed that Cr was the most significantly altered product in the Arg and proline metabolism signaling. The level of Cr, which is beneficial for improving muscle strength,^37^ was 8.28-fold greater in mice subjected to DTRF than in mice subjected to ALF (Figure 3c). This finding was corroborated by a coupled enzyme reaction in which the faecal Cr level was detected to increase by 9.24 times after DTRF (Figure 3d). Consistently, DTRF outperformed ALF in increasing mouse grip-force strength (Figure 3e) and faecal Arg contents (Figure 3f).

As Cr functions to accelerate adenosine triphosphate (ATP) regeneration during energy expenditure,^38^ and because the microbial function alteration triggered by DTRF is concentrated on energy production and conversion (Figure 2h), as indicated by Evolutionary genealogy of genes: Non-supervised Orthologous Groups (eggNOG) enrichment in a metagenomic study, we further asked whether the increase in Cr levels was attributed to the enrichment of bacteria in mice subjected to DTRF. To investigate this possibility, we examined the faecal Cr concentration in mice subjected to ALF that received oral administration of *B. pseudolongum*. In accordance with the effect of Arg supplementation, *B. pseudolongum* colonization markedly increased the Cr level *in vivo* (Figure 3g). Meanwhile, *B. animalis* was given in the same way, whereas no significant change was determined for the faecal Cr. An increase in Arg levels was also detected in mice subjected to ALF after treatment with *B. pseudolongum* rather than *B. animalis* (Figure S7, Supporting Information). These data suggest that the microbiota remodeling in mice subjected to DTRF, particularly the enrichment of radioprotective *B. pseudolongum*, can increase the content of Arg metabolite Cr in the gut.

### *Cr mitigates IR-induced intestinal epithelial injury* in vitro *and* in vivo

**Figure 4.**
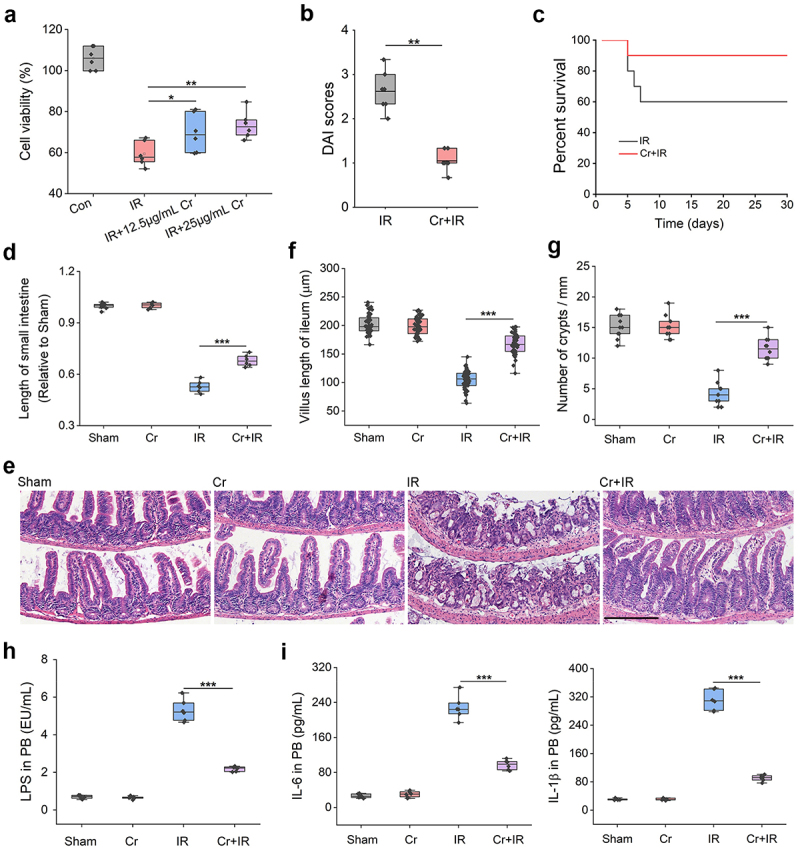
Cr reduces IR-induced intestinal toxicity *in vitro* and *in vivo*. (a) Viability of irradiated IEC-6 cells in the absence or presence of Cr. Results are shown in the boxplot (*n* = 6). *, *p* < 0.05; **, *p* < 0.01. (b) DAI scores of the mice on day 5 after TAI. Results are shown in the boxplot (*n* = 7). ***, *p* < 0.001. (c) Percent survival of the mice after TAI and Cr administration (*n* = 10). (d) Relative length of the mouse small intestine is shown in the boxplot (*n* = 6). ***, *p* < 0.001. (e) HE staining revealing the mitigation of Cr on the intestinal mucosal destruction caused by IR. Scale indicates 200 μm. (f) Measurement of the mouse ileal villous length. Fifty ileal villi were measured for each group. Results are shown in the boxplot. ***, *p* < 0.001. (g) Number of crypts per millimeter in the ileum of mice. Ten fields of view were randomly selected for crypts counting. Results are shown in the boxplot. ***, *P* < 0.001. (h) Determination of the LPS levels in mouse serum. Results are shown in the boxplot (*n* = 6). ***, *p* < 0.001. (i) IL-6 and IL-1β contents in mouse serum. PB, peripheral blood. Results are shown in the boxplot (*n* = 6). ***, *p* < 0.001.

To investigate whether the changed gut microbiota metabolites account for the observed radioprotection, we preincubated rat intestinal epithelial IEC-6 with kinetin 9-riboside, dl-3,4-dihydroxymandelic acid and Cr, three metabolites that were markedly increased in mice subjected to DTRF, and found that only Cr could improve cell survival 24 h after 8-Gy γ-ray IR (Figures 4a and S8, Supporting Information). The radioprotective effect of Cr was also confirmed using human intestinal epithelial HIEC-6 cells (Figure S9, Supporting Information). Furthermore, we added 1% Cr to the drinking water three days before irradiation and repeated the TAI experiment in mice subjected to ALF. The results showed that Cr supplementation relieved the intestinal symptoms caused by TAI (Figure 4b) and improved mouse survival from 60% to 90% (Figure 4c). Additional investigations revealed the mitigation of Cr on the pathological changes in mice small intestine caused by IR, including the tissue shrinkage (Figure 4d), ileal villous shedding (Figure 4e), shortened villous length (Figure 4f) and reduced crypt counts (Figure 4g). The loss of tight junction proteins, claudin-1 and occluding (Figure S10, Supporting Information), in irradiated mice was also inhibited by Cr. As intestinal mucosal destruction enables the invasion of endogenous pathogens and toxins into the circulation, we detected abundant bacteria (Figure S11, Supporting Information) and a high level of endotoxin (LPS, Figure 4h) in the blood on Day 3 after TAI in mice. Owing to the maintenance of barrier integrity, Cr treatment markedly dampened bacterial and LPS translocation, resulting in a prominent reduction in the levels of proinflammatory factors, including IL-6 and IL-1β (Figure 4i). The above results indicate that Cr administration can mitigate IRIII and reduce bacteremia and proinflammatory responses.

### Cr suppresses IR-induced ferroptosis by activating AMPK under energy stress

**Figure 5.**
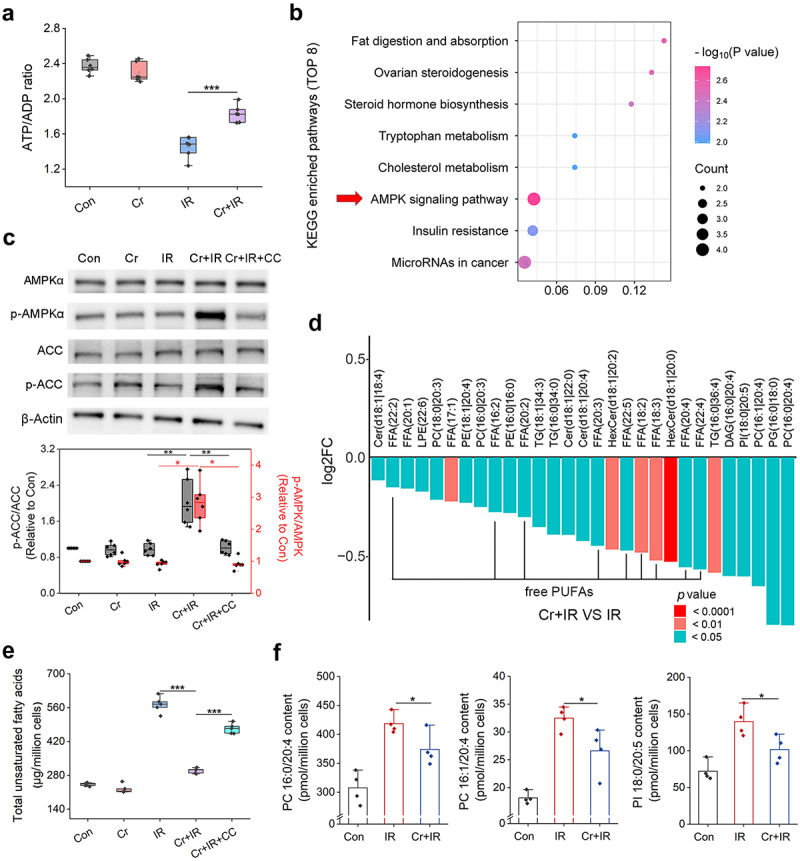
Cr activates AMPK to inhibit PUFAs production caused by IR. (a) Ratio of ATP to ADP in IEC-6 cells in the absence or presence of IR and 25 µg/mL Cr. Results are shown in the boxplot (*n* = 6). ***, *p* < 0.001. (b) Top 8 pathways enriched by KEGG. (c) Protein bands of AMPKα, p-AMPKα, ACC and p-ACC in the cells. β-Actin is the reference. The ratios of p-AMPKα to AMPKα and p-ACC to ACC were measured by gray analysis and shown in the boxplot (*n* = 6). ***, *p* < 0.001. (d) Lipid profile alterations after Cr treatment in irradiated IEC-6 cells. (e) Total unsaturated fatty acids in IEC-6 cells. Results are shown in the boxplot (*n* = 6). ***, *p* < 0.001. (f) Contents of PCs (16:0/20:4 and 16:1/20:4) and PI (18:0/20:5) in IEC-6 cells. Results are shown as the means + SDs (*n* = 4). *, *p* < 0.05.

Because Cr is an important component of the energy shuttle system that buffers ATP and adenosine diphosphate (ADP) levels, we next inferred that the radioprotective effect of Cr might correlate to energy metabolism. Determination of the ATP/ADP ratio supplied that IR (8-Gy γ-ray) caused an energy stress in irradiated IEC-6 cells, whereas this energy deficiency was mitigated by Cr (Figure 5a). Along with energy fluctuations, the status of energy-sensing effectors in irradiated cells might be affected. Our data-independent acquisition (DIA) proteomics study revealed that 96 proteins (FC > 1.5 or FC < 0.67, *p* < 0.05) exhibited altered levels in irradiated IEC-6 cells after treatment with 25 µg/mL Cr (Figure S12, Supporting Information). AMP-activated protein kinase (AMPK) signaling was determined to be the most significantly enriched pathway (*p* = 0.0018) via KEGG analysis of the upregulated proteins (Figure 5b). Western blotting revealed that Cr increased the phosphorylation of AMPKα (p-AMPKα) in the cells after IR (Figure 5c). The downstream effector acetyl-CoA carboxylase (ACC) also presented increased phosphorylation after Cr treatment.

ACC is known a rate-limiting enzyme for de novo lipogenesis.^39^ To investigate the impact of the enhanced phosphorylation of ACC caused by Cr on lipid metabolism, we performed targeted lipidomic analysis in IEC-6 cells with treatment of vehicle (Control), IR (8-Gy γ-ray), or Cr (25 µg/mL)+IR. As shown in Figure 5d, Cr administration significantly decreased the contents of 29 lipids in the cells 24 h after irradiation (*p* < 0.05), including 11 free unsaturated fatty acids (2 monounsaturated fatty acid and 9 PUFAs), which was further confirmed by a lipid assay for total unsaturated fatty acids (Figure 5e). As PUFAs can incorporate into phospholipids, several phospholipids containing PUFAs (PUFA-LPs) that were markedly increased by IR in IEC-6 cells, such as phosphatidylcholines (PCs, 16:0/20:4 and 16:1/20:4) and phosphatidylinositol (PI, 18:0/20:5), were significantly reduced by Cr (Figure 5f, *p* < 0.05).

**Figure 6.**
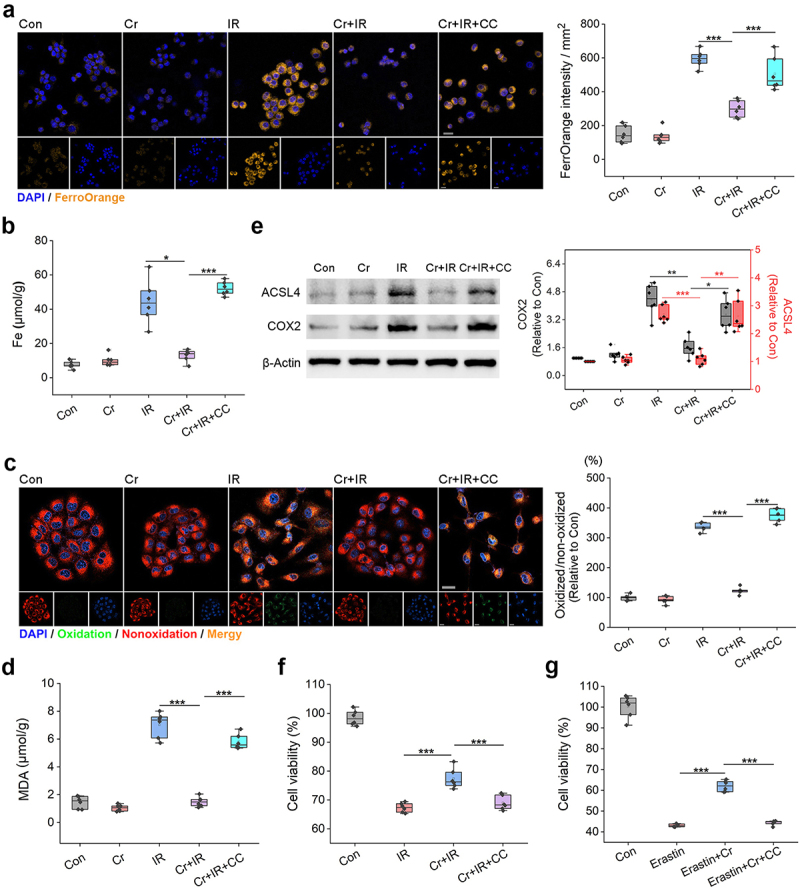
Cr activates AMPK to inhibit ferroptosis caused by IR. (a) Confocal microscopy revealing the Fe^2+^ accumulation (orange) in the cells. Nuclei are stained by DAPI (blue). Scale bar indicates 50 μm. FerrOrange intensity per square milimeter is shown in the boxplot (*n* = 6). ***, *p* < 0.001. (b) Fe contents in the cells. Results are shown in the boxplot (*n* = 6). ***, *p* < 0.001. (c) C11-BODIPY staining indicating the lipid peroxidation in IEC-6 cells. The ratio of the mean fluorescence intensity of oxidized probes to that of non-oxidized probes is shown in the boxplot (*n* = 6). ***, *p* < 0.001. (d) MDA contents in the cells. Results are shown in the boxplot (*n* = 6). ***, *p* < 0.001. (e) Protein bands of ACSL4 and COX2 in the cells. β-Actin is the reference. The ACSL4 and COX2 levels measured by gray analysis are shown in the boxplot (*n* = 6). *, *p* < 0.05; **, *p* < 0.01; ***, *p* < 0.001. (f) Viability of irradiated cells in the absence and presence of 25 µg/mL Cr or 0.5 µM CC. Results are shown in the boxplot (*n* = 6). ***, *p* < 0.001. (g) Viability of IEC-6 cells treated with 5 µM erastin in the absence and presence of 25 µg/mL Cr or 0.5 µM CC. Results are shown in the boxplot (*n* = 6). ***, *p* < 0.001.

Due to the weak C-H bond at the bis-allylic positions, PUFA-LPs are highly vulnerable to peroxidation and are essential for the execution of ferroptosis, a form of programmed cell death driven by iron-dependent lipid peroxidation of PUFA-PLs in cellular membranes.^40,41^ Incubation of IEC-6 and HIEC-6 cells with Fer-1 markedly increased the cell survival 24 h after 8-Gy γ-ray IR (Figure S13, Supporting Information), corroborating that ferroptosis is an important cause of IRIII.^24,26,27^ Accordingly, we speculated that Cr might improve the survival of irradiated enterocytes via suppression of ferroptosis. To verify this hypothesis, we first determined iron contents in the cells. FerroOrange staining revealed an increased level of unstable divalent iron ions (Fe^2+^) in IEC-6 cells after IR, whereas the Fe^2+^ accumulation was markedly attenuated by 25 µg/mL Cr (Figure 6a, *p* < 0.001). Determination of the total Fe content in the irradiated cells also confirmed the ability of Cr to mitigate Fe accumulation (Figure 6b, *p* < 0.05). C11-BODIPY581/591 staining was additionally performed and Cr was discovered to significantly decrease the lipid peroxidation in the irradiated cells (Figure 6c, *p* < 0.001), which was subsequently corroborated by measuring the content of malondialdehyde (MDA), a product of cellular lipid peroxidation (Figure 6d, *p* < 0.001). The levels of acyl-CoA synthetase long-chain family member 4 (ACSL4) and cyclooxygenase-2 (COX2), two ferroptosis-related indicators that were upregulated in IEC-6 after IR, were also repressed by Cr, as indicated by Western blotting (Figure 6e). Additionally, the application of 0.5 µM compound C (CC), a small molecule inhibitor of AMPK,^42^ counteracted the ability of Cr to suppress the activation of AMPK/ACC signaling (Figure 5c), unsaturated fatty acid production (Figure 5e), Fe accumulation, lipid peroxidation, and increase in cellular ACSL4 and COX2 (Figure 6a-e), resulting in an abrogation of the radioprotective effect of Cr (Figure 6f). CC administration also markedly compromised the mitigation of Cr on the death of IEC-6 (Figure 6g, *p* < 0.001) and HIEC-6 cells (Figure S14, Supporting Information) caused by 5 µM erastin, which is an acknowledged ferroptosis activator.^43^ These findings demonstrate that Cr has an inhibitory effect on IR-induced ferroptosis by reducing PUFAs production and that this process is dependent on AMPK activation.

### Cr contributes to the ferroptosis inhibition and radioprotection of DTRF

**Figure 7.**
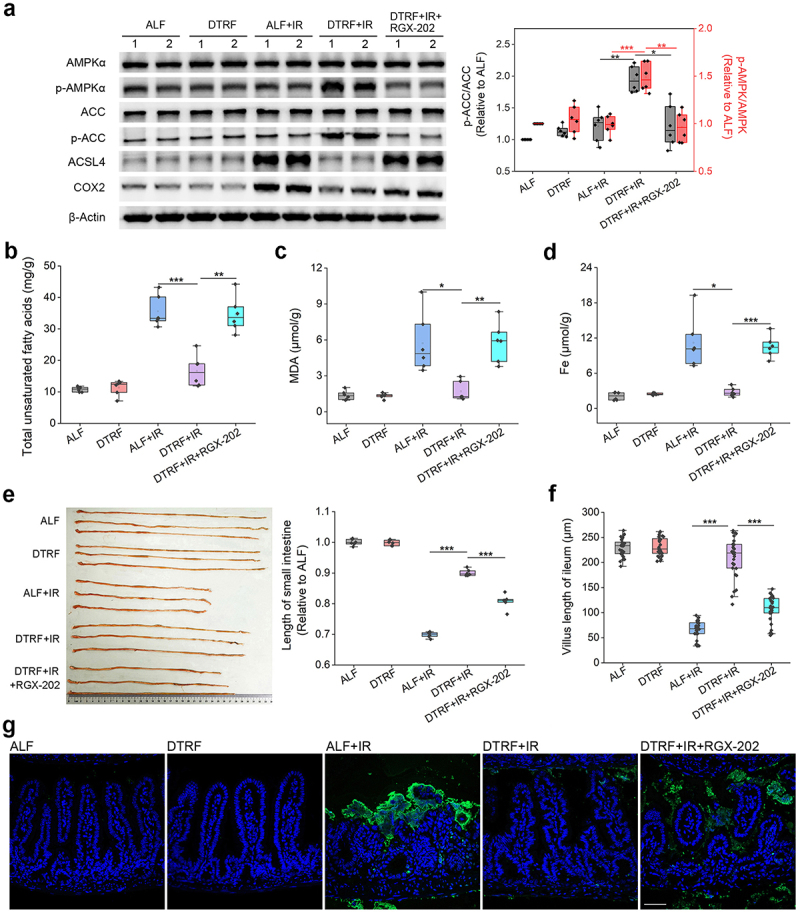
Cr contributes to the radioprotection of DTRF. (a) Protein bands of AMPKα, p-AMPKα, ACC, p-ACC, ACSL4 and COX2 in mice ileum. β-Actin is the reference. The protein levels measured by gray analysis are shown in the boxplot (*n* = 6). *, *p* < 0.05; **, *p* < 0.01; ***, *p* < 0.001. (b) Total unsaturated fatty acids in mice ileum. Results are shown in the boxplot (*n* = 6). **, *p* < 0.01; ***, *p* < 0.001. (c) MDA contents in mice ileum. Results are shown in the boxplot (*n* = 6). *, *p* < 0.05; **, *p* < 0.01. (d) Fe contents in mice ileum. Results are shown in the boxplot (*n* = 6). *, *p* < 0.05; ***, *p* < 0.001. (e) Representative photograph showing the small intestine shrinkage in irradiated mice. Relative length of the mouse small intestine is shown in the boxplot (*n* = 6). ***, *p* < 0.001. (f) Measurement of the villous length in mice ileum. Fifty ileal villi were measured for each group. Results are shown in the boxplot. ***, *p* < 0.001. (g) TUNEL staining revealing the impact of RGX-202 administration on the radioprotection of DTRF. Dead cells are indicated by green fluorescence. Nuclei are stained by DAPI (blue). Scale indicates 50 μm.

To investigate whether Cr-mediated ferroptosis inhibition contributes to the radioprotection of DTRF, we further performed the mouse TAI experiment and evaluated the influence of 3-Guanidinopropionic acid (RGX-202), an oral small-molecule solute carrier family 6 member 8 (SLC6A8) transporter inhibitor that suppresses Cr import *in vivo*.^44^ Consistent with the findings in the irradiated cells treated with Cr, DTRF increased p-AMPKα and p-ACC levels (Figure 7a) but decreased ACSL4 and COX2 expressions (Figure S15, Supporting Information) in the ileum in mice that received TAI. Compared with those in mice subjected to ALF, decreased levels of unsaturated fatty acid production (Figure 7b, *p* < 0.001), lipid peroxidation (Figure 7c, *p* < 0.001) and Fe accumulation (Figure 7d, *p* < 0.001) were also detected in mice subjected to DTRF after irradiation. Nevertheless, these improvements elicited by DTRF were abrogated by the oral administration of RGX-202. Measurement of the length of the small intestine revealed a marked reduction in tissue shrinkage in irradiated mice after DTRF (Figure 7e, *p* < 0.001), which was supported by histopathological analysis, in which the villous shortening (Figure 7f, *p* < 0.001), loss of intestinal crypts (Figure S16, Supporting Information), and epithelial cell death (Figure 7g) induced by IR were abated by DTRF. However, RGX-202 administration impaired the ability of DTRF to maintain intestinal length and mucosal barrier integrity after TAI (Figures 7e-g).

Because *B. pseudolongum* enrichment increased the intestinal Cr content and contributed to the radioprotective effect of DTRF, we also evaluated the influence of RGX-202 administration on the improvement of this probiotic. Similar to DTRF, *B. pseudolongum* supplementation ameliorated the pathological changes, including villous shortening and loss of intestinal crypts, and reduced the production of unsaturated fatty acid, lipid peroxidation and Fe accumulation in the ileum of irradiated mice (Figure S17, Supporting Information). Nevertheless, the radioprotection caused by *B. pseudolongum* was significantly attenuated by RGX-202 treatment. These findings underpin that ferroptosis inhibition induced by Cr is involved in the mitigation of DTRF on IRIII.

## Discussion

The gut microbiota dysbiosis and ferroptosis in intestinal epithelial cells (IECs) are important causes driving the pathogenesis of IRIII.^6,8,27^ It has been discovered that regulation of the gut microbiota can reduce IR-induced intestinal toxicity.^3,7^ TRF is an emerging behavioral nutrition approach affecting the gut microbiota composition.^15,16^ Whether this dietary pattern influences the intestinal radiosensitivity is unclear. Furthermore, the gut microbiota has regulatory effects on ferroptosis,^28^ raising a possibility that ferroptosis inhibition may contribute to the radioprotection of gut microbiota regulation. Herein, we evaluated the impact of TRF on intestinal radiosensitivity and demonstrated that DTRF could reshape the gut microbiota to mitigate IRIII, to which the ferroptosis inhibition caused by intestinal Cr contribute.

Our metagenomic study revealed that *B. pseudolongum*, a predominant Bifidobacterial species in various mammals,^45^ was associated with the radioprotective effect of DTRF. *B. pseudolongum* has long been known to ameliorate severe radiation injury,^36^ but the underlying mechanism remains vague. We discovered that *B. pseudolongum* enabled to increase the enteric Arg content and facilitate the production of Cr, which protected IECs from lethal IR. As known, endogenous Arg can be synthesized *de novo* through the consumption of glutamate and acetyl-CoA in many microorganisms.^46^ Since *B. pseudolongum* generates acetate that can be transformed into acetyl-CoA,^47^ we propose that this bacterium may promote Arg production by supplying extra acetyl-CoA. Intriguingly, because increased intestinal Arg levels facilitate the colonization of *B. pseudolongum*,^48^ there seems to be a positive feedback loop related to Arg metabolism after DTRF, accounting for the observed increase in Cr abundance. Cr is a nitrogenous organic compound that promotes improvements in muscle and exercise performance.^38^ The finding that *B. pseudolongum* increased enteric Cr levels corroborated the predominant shift in microbial function to energy production and conversion in mice subjected to DTRF and underpinned the efficacy of *Bifidobacterium* supplementation in restoring age-related muscle loss.^49^

The study is the first, to our knowledge, to identify Cr as a ferroptosis inhibitor to achieve radioprotection. Mechanistically, Cr activated AMPK and induced the phosphorylation of downstream ACC, resulting in reduced biosynthesis of unsaturated fatty acids, which provides the substrates for lipid peroxidation during ferroptosis.^22^ A similar mechanism was revealed for the suppression of ferroptosis by energy stress.^40^ AMPK inhibition abrogated the alleviation of IR-induced ferroptosis by Cr, which supports the role of AMPK activation in IRIII mitigation.^27^ Moreover, we found that Cr could markedly improve the survival of cells exposed to ferroptosis inducer erastin, possibly due to that AMPK activation negatively regulates ferroptosis induced by erastin.^50^

AMPK is a pivotal sensor of cellular energy status. In the presence of decreasing cellular energy levels, AMPK activation switches on catabolism to generate ATP while downregulating anabolism to reduce ATP consumption.^51^ IR induced energy stress in IECs. However, neither we nor the authors of a recent study reported the activation of AMPK in IECs after IR,^27^ which suggested the occurrence of energy metabolism maladaptation. The ability of Cr to increase AMPK activation after IRIII indicated the ability of this metabolite to facilitate metabolic adaptation under energy stress. Cr is taken up by IECs through the SLC6A8 transporter and is transformed to phosphorylcreatine (PCr) under the catalysis of Cr kinase. An increased ratio of cellular Cr to PCr induces the activation of AMPK.^52^ Because SLC6A8 expression is upregulated by IR,^53^ more Cr might be transported to the cytoplasm, which may explain why Cr triggered more marked AMPK activation in IECs after irradiation injury than in normal cells.

Cr is widely used by healthy individuals and patients with neurological disorders. It possesses a diverse set of regulatory effects beyond the effects of a typical energy supplement. A pioneering study revealed that Cr could increase energy availability in T cells partially through AMPK activation to eliminate cancer cells.^54^ As small intestinal injury is a primary obstacle for patients with abdominal and pelvic tumors who are undergoing high-dose radiotherapy, Cr supplementation may protect the small intestine from IR while enhancing the efficacy of tumor radiotherapy in these patients. Notably, emerging studies have indicated a role for Cr in facilitating cancer progression and metastasis,^44,55^ which highlights the delicate balance of Cr in cancers^56^ and raises questions about the impacts of Cr supplementation on the outcome of radiotherapy. Currently, there is no clear correlation between enteric Cr levels and tumor metastasis after radiotherapy. Considering the promising radioprotective effect of Cr on normal intestinal tissues, further investigations to identify reasonable Cr supplementation schemes to reduce IR-induced intestinal toxicity without compromising the prognosis of tumor radiotherapy are needed. Alternatively, inhibiting Cr uptake by cancer cells through tumor-targeting technology may be effective.^57^ Additionally, since circadian reprogramming explains the time-of-day effect of TRF,^58^ whether the increased Cr levels in mice subjected to DTRF are dictated by circadian regulation also requires further exploration.

**Figure 8.**
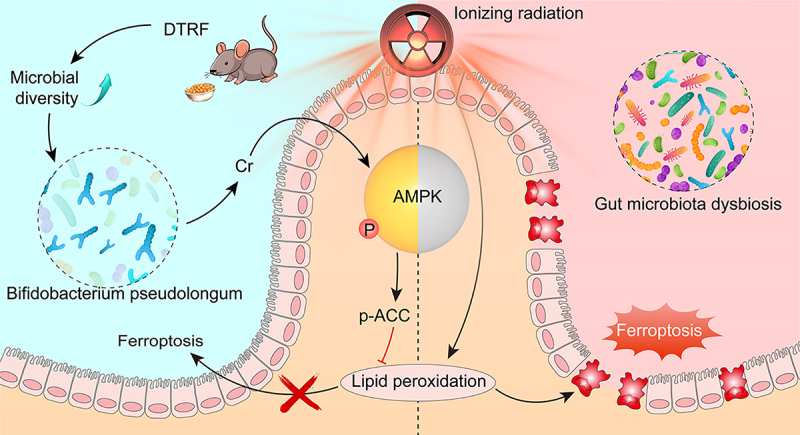
Schematic illustration of the radioprotective effect of DTRF. DTRF reshapes the gut microbiota and enriches *B. pseudolongum*, which increases the intestinal Cr levels. Cr promotes metabolic adaptation by activating AMPK, which induces the phosphorylation of ACC and results in reduced lipid peroxidation and reduced ferroptosis, thereby achieving the intestinal radioprotection.

Overall, our findings show that DTRF enriches *B. pseudolongum* and increases the level of enteric Cr, which can reduce IR-induced intestinal toxicity. Under energy stress caused by IR, Cr promotes metabolic adaptation by activating AMPK, which induces phosphorylation of the downstream effector ACC, resulting in reduced lipid peroxidation and reduced ferroptosis (Figure 8). Given that the effective measures for mitigating IRIII are lacking, we believe that in addition to DTRF, *B. pseudolongum* and Cr supplementation may be important tools for radioprotection. Considering the ferroptosis inhibition of Cr, it may also has application values in the treatment of ferroptosis-related diseases, such as ischemia/reperfusion, Alzheimer’s disease, and Parkinson’s disease.^59^

## Experimental section

### Total abdominal irradiation mouse experiment

Six- to eight-week-old male C57BL/6J mice weighing 18–20 g were purchased from Beijing Vital River Laboratory Animal Technology Co., Inc. (Beijing, China). The mice were housed in a specific pathogen-free facility at the Institute of Combined Injury of PLA, Army Medical University (AMU), and were maintained under controlled conditions (ambient temperature: 22 ± 2°C, air humidity: 40–70%) with a 12‒h light‒dark cycle. After one week of adaptive feeding, the mice were randomly divided into 6 groups (ALF, ALF+IR, DTRF, DTRF+IR, NTRF, and NTRF+IR; *n* = 10 per group). The mice subjected to ALF had continuous access to a standard diet and drinking water. In the DTFR group, the mice were fed only during the ZT2–10 period, while water intake was unlimited throughout the day. For the NTRF mice, feed was provided during the ZT14–22 period, and a sufficient water supply was provided. Mice subjected to ALF, DTRF, or NTRF for 1, 2 or 3 weeks were anaesthetized with 150 µL of 1% pentobarbital sodium and placed in the supine position, followed by TAI at ZT1 using a ^60^Co source of 11 Gy γ-rays at a dosage rate of 0.48 Gy/min.^3^

For the dirty cage experiment, 10 mice subjected to DTRF for one week were transferred to the cages of mice subjected to ALF (*n* = 10) at ZT2. This hybrid cage feeding continued until ZT10, when the mice subjected to DTRF were returned to their previous cages. The mice subjected to ALF were subjected to TAI after 7 days of hybrid cage feeding. To evaluate the radioprotective effect of *B. pseudolongum*, a bacterial strain obtained from China General Microbiological Culture Collection Center (CGMCC, No. 1.3005) was cultured in deMan Rogosa Sharpe (MRS) broth (OXOID, Basingstoke, Hants, England) at 37°C under anaerobic conditions. After resuspension with sterilized PBS, a total of 2 × 10^8^ CFU of *B. pseudolongum* (200 µL) was delivered to mice subjected to ALF (*n* = 10) through oral administration. Probiotic supplementation was performed for 7 consecutive days (q.d.) before TAI. The addition of *B. pseudolongum* was conducted for another 3 days after TAI. Additionally, to evaluate the role of Cr supplementation in TAI, 40 mice subjected to ALF were divided into 4 groups (sham, Cr, IR, and Cr+IR; *n* = 10 per group). Cr (1%; C3630, Sigma-Aldrich, Merck, Shanghai, China) was added to the drinking water 4 days before TAI and continued for 7 days. The mice in the sham group were treated with sterile water. The mice were cared for and treated in accordance with the recommendations of the National Institutes of Health (NIH) Guide for the Care and Use of Laboratory Animals (NIH Publication No. 85e23 Rev. 1985). The animal experiments were approved by the Institutional Review Board of AMU (approval no. AMUWEC20230331). After TAI, mouse survival was monitored for 30 days. DAI scores were determined as previously described on Day 5 post-irradiation.^60^ The specific criteria for DAI scoring is shown in Table S4 (Supporting information).

### Histopathologic analysis

Another 120 mice randomly divided into 20 groups (ALF, ALF+IR, NTRF, NTRF+IR, DTRF, DTRF+IR; sham, Cr, IR, and Cr+IR; ALF, ALF+IR, DTRF, DTRF+IR, and DTRF+IR+RGX-202; sham, IR, *B. pseudolongum*, *B. pseudolongum*+IR, and *B. pseudolongum*+IR+RGX-202; *n* = 6 per group) were used to repeat the TAI experiment. RGX-202 (800 mg/kg; HY-W015828, MedChemExpress, Shanghai, China) was orally administrated 4 days before TAI and continued for 7 days (q.d.). The mice were euthanized 3 days after TAI via the injection of an excessive amount of pentobarbital sodium, and their small intestine tissues were obtained via surgery. Ileum tissues were fixed in Carnoy’s fluid (G1120, Servicebio, Wuhan, China) at 4°C for 48 h, processed with gradient concentrations of ethanol for dehydration, and embedded in paraffin. The samples were sectioned at a 3 µm thickness and observed after hematoxylin and eosin (H&E) staining, after which the ileal villous height and the number of crypts were determined.

For immunofluorescence staining, the paraffin sections were deparaffinized with dimethylbenzene and ethyl alcohol and subjected to antigen retrieval in citrate buffer (10 mM, pH 6.0) at 95 − 100°C for 15 min. Bovine serum albumin (BSA, 1%) was used to block nonspecific epitopes. The sections were then incubated with a rabbit monoclonal anti-occludin antibody (ab216327, Abcam, 1:100) and a rabbit monoclonal anti-claudin 1 antibody (ab307692, Abcam, 1:100). A goat anti-rabbit Alexa Fluor 488 (A0423, Beyotime, Shanghai, China; 1:500) antibody or goat anti-rabbit Alexa Fluor 647 (ab150079, Abcam, 1:500) antibody was used to visualize the locations of the tight junction proteins. Terminal deoxynucleotidyl transferase-mediated dUTP-biotin nick end labeling (TUNEL) staining was performed using a kit from Elabscience (E-CK-A321; Shanghai, China). Images were captured with a Zeiss LSM 780 NLO confocal microscope. Quantitative analysis on the fluorescence intensity was conducted via ZEN 2011 (blue edition).

### Faecal microbiota transplantation

Faecal samples from mice subjected to DTRF were collected after two weeks and resuspended in sterile PBS (1 g/10 mL). The supernatant was obtained after centrifugation at 3000 rpm for 30 s, followed by high-speed centrifugation (12,000 rpm) at 4°C for 5 min to prepare the microbiota pellets, which were further resuspended in 2.5 mL of sterile PBS and delivered to mice subjected to ALF by oral administration (q.d.). The mice were treated with antibiotics containing ampicillin (1 mg/mL; A600064–0025, BBI Life Science, Shanghai, China), metronidazole (1 mg/mL; A6000633–0025, BBI Life Science), neomycin (0.5 mg/mL; A610366–0025, BBI Life Science), and vancomycin (0.5 mg/mL; V105495, Aladdin, Shanghai, China) to eliminate intestinal bacteria 7 days before transfer of the DTRF faecal microbiota. After 7 days of FMT (q.d.), the mice were subjected to TAI, and FMT was further performed for another 3 days. Mouse survival, body weight, and DAI scores were monitored as described for the TAI mouse experiment.

### 16S rRNA gene amplicon sequencing analysis

Fresh faeces from mice treated with ALF or DTRF (*n* = 10) for two weeks were obtained and stored at −80°C until use. Faecal samples were also collected from mice subjected to ALF and DTRF (*n* = 6) 48 h after TAI. DNA from the stool was extracted via a TIANGEN magnetic soil and stool DNA kit (4992736, Beijing, China). The polymerase chain reaction (PCR) products were purified with a Qiagen Gel Extraction Kit (28706, Germany). 16S ribosomal RNA (rRNA) V4 was amplified using specific primers and Phusion High-Fidelity PCR Master Mix (M0531L, New England Biolabs, Beijing, China). The TIANGEN Universal DNA Purification Kit (DP214) was used to purify the PCR products. Library construction, sequencing, amplicon sequence variant (ASV) denoising and species annotation were performed by Novogene (Beijing, China). The sequencing libraries were generated via the NEBNext® Ultra^™^ II FS DNA Library Prep kit (E6177S, New England Biolabs) and evaluated via a Qubit@ 2.0 fluorometer (Thermo Fisher Scientific, Shanghai, China).

High-throughput gene sequencing was conducted on a Illumina NovaSeq6000 platform, and 250 bp paired-end reads were obtained, which were further merged via FLASH (version 1.2.11, http://ccb.jhu.edu/software/FLASH/) after the barcode and primer sequences were removed. The raw tags were strictly filtered with fastp (version 0.23.1, https://github.com/OpenGene/fastp#) to generate high-quality clean tags. After comparison with the reference database (Gold database, http://drive5.com/uchime/uchime_download.html) through the UCHIME algorithm (http://www.drive5.com/usearch/manual/uchime_algo.html), the chimaera sequences were selected and removed. Quantitative Insights Into Microbial Ecology 2 (QIIME2) employs Divisive Amplicon Denoising Algorithm (DADA2) in the construction of ASVs.^61^ Species information for each ASV was obtained by comparing the obtained ASVs with the database via the classify-sklearn module in QIIME2. Bacterial diversity analysis was performed on the NovoMagic platform (https://magic.novogene.com). The indices, including Observed OTUs, Shannon, Simpson, Chao 1, Goods coverage, Dominance, and Pielou E were applied to analyze microbial α diversity. PERMANOVA was performed to evaluate microbial β-diversity (weighted UniFrac metrics). A t test was used to analyze species differences at different levels.

### Metagenomic analysis

Fresh faecal samples from mice subjected to ALF or DTRF (*n* = 6) for two weeks were collected and stored at −80°C until use. DNA from the faeces, which was extracted as described for bacterial diversity analysis, was broken into short fragments. Before size selection, these fragments were end-polished, subjected to the addition of poly A tails, and ligated with full-length adapters for Illumina sequencing. An Agilent (Beijing, China) 2100 bioanalyzer was used to evaluate the insert size of the library. The Q‒PCR method was used to quantify the effective concentration of the library accurately (effective concentration >3 nM). The qualified libraries were pooled and sequenced on the Illumina platform PE150 at Novogene (Beijing, China). The raw data were processed with fastp (version 0.23.1) to generate clean data, which were blasted against the mouse genome (GRCm38) to filter out reads that may have originated from the host. The clean data were further assembled via MEGAHIT software with the following parameter settings: –presets meta-large (–end-to-end, –sensitive, -I 200, -X 400).^62,63^ ORF prediction for scaftigs (≥500 bp) was performed for each sample via MetaGeneMark (http://topaz.gatech.edu/GeneMark/). CD-HIT software (http://www.bioinformatics.org/cd-hit/) was used to eliminate redundancy and collect the nonredundant initial gene catalog. To calculate the number of reads of the genes in each sample alignment, the clean data were aligned to the initial gene catalog via Bowtie2.^64^ After filtering out genes with ≤ 2 reads in each sample, the final gene catalog for subsequent analysis was obtained. For species annotation, DIAMOND software (https://github.com/bbuchfink/diamond/) was employed for alignment of unigene sequences with the Micro_NR database. The species annotation information of the sequence was determined via the LCA algorithm, and diversity analysis was performed on the NovoMagic platform (https://magic.novogene.com). LEfSe software was used for LEfSe analysis (LDA score of 4 by default). Functional annotations were performed using DIAMOND software to align unigenes with those in the eggNOG database (http://eggnogdb.embl.de/#/app/home).

### Untargeted metabolomics

Faecal samples (100 mg for each) from mice treated with ALF or DTRF (n = 6) for two weeks were ground with liquid nitrogen, followed by resuspension in 500 µL of prechilled 80% methanol. After coincubation on ice for 5 min, the homogenate was centrifuged at 15,000 × g and 4°C for 20 min. The supernatant was diluted with liquid chromatography‒mass spectrometry (LC‒MS)-grade water (1.15333.2500, Merck) to generate a solution containing 53% methanol and centrifuged for further purification. Ultrahigh-performance liquid chromatography‒tandem mass spectrometry (UHPLC‒MS/MS) analysis of the purified supernatant was then conducted via a Vanquish UHPLC system (Thermo Fisher) coupled with an Orbitrap Q ExactiveTM HF mass spectrometer (Thermo Fisher) at Novogene (Beijing, China). A Hypersil Gold column (C18, 100 × 2.1 mm, 1.9 μm; Thermo Fisher) and a 12-min linear gradient at a flow rate of 0.2 mL/min were applied for sample analysis. Eluents A and B were 0.1% formic acid (FA) in water and methanol, respectively. Mass spectrometry was performed in positive/negative polarity mode with a spray voltage of 3.5 kV, capillary temperature of 320°C, S-lens RF level of 60, and Aux gas heater temperature of 350°C. The raw data were processed with Compound Discoverer 3.3 (CD3.3, Thermo Fisher) to perform peak alignment and selection and metabolite quantification. Statistical analysis was performed using R (v.*R*-3.4.3), Python (Python, v.2.7.6) and CentOS (v.6.6) software. The obtained metabolites were annotated using the KEGG database (https://www.genome.jp/kegg/pathway.html), HMDB (https://hmdb.ca/metabolites) and LIPIDMaps database (http://www.lipidmaps.org/). Metabolites with VIP > 1, *p* value < 0.05 and FC > 2 or FC < 0.5, were considered to be significantly altered. The functions of the identified metabolites and metabolic pathways were investigated via the Kyoto Encyclopedia of Genes and Genomes (KEGG, https://www.genome.jp/kegg/) database.

### Cr and Arg determination

Fresh faeces from mice subjected to ALF or DTRF (*n* = 6) for two weeks, and from mice subjected to ALF received oral administration of 2 × 10^8^ CFU of *B. pseudolongum* and *B. animalis* (200 µL, q.d.) for 7 consecutive days (*n* = 6), were collected and homogenized in sterile PBS (20 mg/500 µL). *B. animalis* was obtained from CGMCC (No. 1.2268) and cultured in MRS broth at 37°C under anaerobic conditions. The supernatant was obtained after centrifugation at 15,000 × g and 4°C for 10 min. Cr contents were determined using a Cr assay kit (MAK079, Sigma Aldrich, Shanghai, China). Fifty microliters of each sample was coincubated with a reaction mixture containing Cr assay buffer, creatinase, and the Cr probe (50 µL in total) at 37°C for 60 min. Absorbance at 570 nm was then detected using a Molecular Devices M2e microplate reader (Sunnyvale, CA, USA). For Arg determination, the amount of the supernatant derived from the faecal homogenate was measured using an Arg content assay kit (BC5635, Solarbio) according to the manufacturer’s instructions.

### Mouse grip-force strength determination

The grip force of mice subjected to ALF and DTRF (*n* = 6) was assessed with a 47200 Grip Strength Meter (Ugo Basile, Italy). The mice were placed on a metal grid so that their paws were gripping the wire mesh grid, which equipped with a force transducer. Subsequently, the mice were pulled by the tail with increasing force until they release their claws. The maximum grip force was recorded for each mouse after 6 assessments. The same person conducted this experiment.

### Evaluation of bacteraemia and endotoxaemia

Fresh blood from the hearts of mice subjected to TAI in the presence or absence of Cr supplementation was diluted 10 times in sterile PBS. One hundred microliters of the sample was used to evenly coat Mueller Hinton agar and cultured in a 37°C constant-temperature incubator. Bacterial colonies were counted after 24 h. For evaluation of endotoxaemia, the blood samples were centrifuged at 3000 rpm for 15 min to collect the serum, one hundred microliters of which was further coincubated with 100 µL of tachypleus amebocyte lysate (EC80545, Xiamen Bioendo Technology, Fujian Province, China) at 37°C for 10 min. Afterward, one hundred microliters of the chromogenic matrix solution was added and incubated for 6 min. Hydrochloric acid solution was added to stop the reaction. The absorbance of the mixture at 545 nm was finally measured after the addition of the azo reagent using a microplate reader. IL-6 and IL-1β levels in the serum were determined via enzyme-linked immunosorbent assay (ELISA), in which two Beyotime kits (PI301 and PI326) were used according to the manufacturer’s instructions.

### Cell experiments

IEC-6 (CRL-1592) and HIEC-6 (CRL-3266) cells were obtained from the American Type Culture Collection (ATCC) and cultured in Dulbecco’s modified Eagle’s medium (DMEM; Gibco, Thermo Fisher) supplemented with 10% fetal bovine serum (FBS; Gibco). The cells were grown at 5% CO_2_ and 37°C, and the number of passages was no more than 8 during the experiment. Cells were seeded into sterile 96-well plates at a density of 5000 CFU/well and cultured overnight. IR was performed using a ^60^Co source of 8 Gy γ-rays at a dose rate of 0.48 Gy/min. Cr (12.5 and 25 µg/mL), kinetin 9-riboside (T7337, TargetMol, Shanghai, China; 5, 10, 20 and 50 nM), dl-3,4-dihydroxymandelic acid (151610, Sigma-Aldrich; 10, 20, 50 and 100 µM), and Fer-1 (S7243, Selleck Chemicals, USA; 10 and 20 µM) were added 6 h before IR, and 5 µM erastin (S7242, Selleck Chemicals) was added 4 h before Cr. Cell viability was assessed 24 h after IR using a cell counting kit-8 (Dojindo, Shanghai, China). To evaluate the influence of AMPK inhibition on the radioprotective effect of Cr, CC (0.5 µM; BML-275, MedChemExpress) was added 1 h before Cr supplementation. These experiments were conducted in duplicate and repeated three times.

Lipids from IEC-6 cells subjected to 8-Gy γ-ray irradiation, in the presence or absence of Cr (25 µg/mL) or 0.5 µM CC 6 h before IR, were extracted using a lipid extraction kit (ab211044, Abcam). Unsaturated fatty acids were measured with a lipid assay kit (ab242305, Abcam). A fluorescent ratio probe for lipid oxidation, C11-BODIPY581/591 (HY-D1301, MedChemExpress), was applied to detect lipid peroxidation. The intracellular Fe^2+^ concentration was probed with 10 μM FerroOrange (F374, Dojindo). Images were captured with a Zeiss LSM 780 NLO confocal microscope. Quantitative analysis was conducted using ZEN 2011 (blue edition). The MDA levels in the cells and ileal tissues were measured via the classic thiobarbituric acid (TBA) method (M496, Dojindo). The total iron content was determined via a colorimetric method (I291, Dojindo). These assays were conducted in duplicate and repeated three times.

### ATP/ADP ratio detection

IEC-6 cells were seeded into a sterile 96-well plate at a density of 1000 CFU/well, cultured overnight at 5% CO_2_ and 37°C, and irradiated with an 8 Gy γ-ray at a dose rate of 0.48 Gy/min. Cr (25 µg/mL) was added 6 h before IR. The ATP/ADP ratio in IEC-6 cells in the absence or presence of IR and Cr after 24 h was detected via a luminescence kit from Dojindo (A552). This assay was conducted in duplicate and repeated three times.

### Proteomics analysis

Proteins from IEC-6 cells in the presence or absence of 25 µg/mL Cr were extracted 24 h after irradiation using a dissolved buffer containing 8 mM urea and 100 mM triethylammonium bicarbonate (TEAB; T7408, Sigma-Aldrich). The supernatant was obtained by centrifugation at 12,000 × g and 4°C. Dithiothreitol (DTT, D9163, Sigma-Aldrich; 1 M) was then added, and the mixture was incubated at 56°C for 1 h, followed by incubation in an ice bath for 2 min. Anhydrous iodoacetamide (IAM, I6125, Sigma-Aldrich) was further added, and the mixture was incubated at room temperature in the dark for 1 h. The protein samples were quantified and digested with trypsin. FA was added to adjust the solution pH to less than 3.0. The supernatant was desalted, washed with a cleanout solution containing 0.1% FA and 3% acetonitrile (ACN) three times, and eluted with a solution containing 0.1% FA and 70% ACN.

The samples were then analyzed using the Vanquish™ Neo UHPLC system (Thermo Fisher) equipped with a C18 precolumn (174500, 5 mm × 300 µm, 5 µm; Thermo Fisher) and a C18 analytical column (ES906, 150 µm × 15 cm, 2 µm; Thermo Fisher). Mass spectrometry was conducted at Novogene Co., Ltd. (Beijing, CHN) using a Thermo Orbitrap astral mass spectrometer equipped with an easy spray ion source. The ion spray voltage was set to 1.9 kV, and the mass spectrum was collected in data-dependent collection mode. The full scan range of the primary mass spectrum was 380–980 (m/z), and the resolution of the primary mass spectrum was 240,000 (200 m/z). The secondary m/z collection range was 150 to 2000. The raw data obtained by mass spectrometry were analyzed using DIA-NN software.^65^ Peptide segments and proteins with false discovery rates (FDRs) above 1% were removed. Proteins with *p* values < 0.05 and FC > 1.5 or FC < 0.67 were considered to be significantly altered. The functional enrichment of the differentially expressed proteins was analyzed via the KEGG.

### Western blotting

IEC-6 cells and mice ileal tissues were collected and lysed for protein extraction. Twenty-five micrograms of each sample were resolved via 10% sodium dodecyl sulfate‒polyacrylamide gel electrophoresis. The detailed information for the antibodies used to detect AMPKα, p-AMPKα, ACC, p-ACC, ACSL4 and COX2 were shown in Table S5 (Supporting Information). A BeyoECL Plus chemiluminescence kit (P0018S, Beyotime) and a Bio-Rad ChemiDoc^TM^ MP Imaging System (Hercules, CA, USA) were used to visualize the protein bands. β-Actin was employed as a reference.

### Lipidomic analysis

Lipids from IEC-6 cells in the presence or absence of 25 µg/mL Cr were extracted 24 h after 8-Gy γ-ray IR. The targeted lipidomic analysis was carried out in Metabo-Profile Biotechnology (Shanghai, China) as previously described.^66^ Briefly, the cell samples were thawed in an ice bath. Isopropanol (250 µL) and 10 magnetic beads were added to each tube of samples, followed by a homogenization using a Next Advance Bullet Blender Homogenizer (BB24; Averill, NY, USA) for 3 min. Samples were kept at −20°C for 10 min and then vortexed for 10 min using an MSC-100 constant temperature mixer (Allsheng Instruments, Hangzhou, China). The supernatant was collected after 2 h by centrifugation at 18,000 g for 10 min and processed for LC-MS analysis.

Lipids were separated using an Acquity UPLC BEH amide column (2.1 × 100 mm, 1.7 μm) on Acquity UPLC I-Xevo TQ-S system (Waters, MA, USA). Mobile phase A was acetonitrile/water (95:5, v/v) containing 5 mM ammonium acetate. Mobile phase B was consisted of acetonitrile/water (50:50, v/v) containing 5 mM ammonium acetate. The gradient profile was 0.1–20% B for 2 min, 20–80% B in 2–5 min, 80–0.1% B in 5–5.1 min, and 0.1% B in 5.1–8 min. The temperature was set at 45°C, and the flow rate was 600 μL/min. Blank and QC samples were injected before and after the analysis of each batch of samples. For mass spectrometry analysis, the capillary voltage of 1.9 and 2.8 kV was used for negative and positive electrospray ionization (ESI) mode, respectively. The source and desolvation temperature were set at 120 and 500°C, respectively. The flow rate of desolvation gas (nitrogen) was 1000 L/h. Raw data files were processed by MassLynx software (Waters, V4.1). The abundance of lipids was quantified by a standard curve.

### Statistical analysis

The significance of differences was analyzed with SPSS 25.0 via one-way analysis of variance (ANOVA) for multiple comparisons or an unpaired two-tailed Student’s t test for comparison between two groups. A *p* value <0.05 was considered to indicate statistical significance.

## Supplementary Material

Supplemental Material

## Data Availability

All data generated or analyzed during this study are included in this published article and its supporting information files. Raw microbial sequencing data are deposited at the NCBI Sequence Read Archive (SRA) under the accession numbers: PRJNA1133968 (16S rRNA, before TAI), PRJNA1134280 (16S rRNA, after TAI), and PRJNA1134918 (Metagenomic).

## Preprint

The manuscript has been shown as a preprint at ResearchSquare (https://doi.org/10.21203/rs.3.rs-4817068/v1).
