# Supplementary material for: Creatine-mediated ferroptosis inhibition is involved in the intestinal radioprotection of daytime-restricted feeding
Source: Gut Microbes. 2025 Apr 9;17(1):2489072. doi: 10.1080/19490976.2025.2489072 (PMC11988229; doi:10.1080/19490976.2025.2489072)
Supplement: Supplemental Material [file KGMI_A_2489072_SM9941.docx]

**Supporting Information**

**Creatine****-mediated ferroptosis inhibition is involved in the intestinal radioprotection of daytime-restricted feeding**

Yingjuan He^a#^, Gaomei Zhao^a#^, Xue Ouyang^a#^, Shaobo Wang^b^, Yin Chen^a^, Chenwenya Li^a^, Yongwu He^a^, Jining Gao^a^, Songling Han^a^, Jinghong Zhao^b^, Junping Wang^a,*^, Cheng Wang^a,*^

^a^ State Key Laboratory of Trauma and Chemical Poisoning, Institute of Combined Injury of PLA, College of Preventive Medicine, Army Medical University, Chongqing, China.

^b^ Department of Nephrology, Xinqiao Hospital, Army Medical University, Chongqing, China.

^#^ Y.H., G.Z., and X.O. contribute equally to this study.

^*^Corresponding Authors

Prof. Cheng Wang

E-mail: wangcheng@tmmu.edu.cn; ORCID: 0000-0002-6690-6433

Prof. Junping Wang

E-mail: [wangjunping@tmmu.edu.cn](mailto:wangjunping@tmmu.edu.cn); ORCID: 0000-0001-5905-0940

**Number of pages**: 15

**Number of tables**: 5

**Number of figures**: 17

**Contents:**

**Table S1**. Relative abundance of top 10 bacterial species at the phylum level

**Table S2**. Significantly changed bacteria in mice subjected to DTRF compared with that in mice subjected to ALF

**Table S3**. Top 8 pathways enriched by KEGG based on the metabolomics results obtained in the positive ion mode

**Table S4.** The specific criteria for DAI scoring

**Table S5**. Detailed information for the antibodies used in Western blotting

**Figure S1**. Food intake and body weight of mice subjected to ALF, DTRF or NTRF for 3 weeks

**Figure S2**. Percent survival of mice subjected to ALF, DTRF or NTRF for 1 and 3 weeks after TAI

**Figure S3**. HE staining showing the ileum tissues of mice subjected to DTRF and NTRF

**Figure S4**. Number of crypts per millimeter in the ileum of mice subjected to ALF, DTRF, or NTRF

**Figure S5**. DTRF improves microbial diversity in irradiated mice

**Figure S6**. Clustering heat map showing the alteration of gut microbiota metabolites in mice after DTRF

**Figure S7**. Faecal Arg contents in mice subjected to ALF after treatment with *B. animalis* or *B. pseudolongum*

**Figure S8**. Influences of kinetin 9-riboside and dl-3,4-dihydroxymandelic acid on the survival of irradiated IEC-6 cells

**Figure S9**. Viability of irradiated HIEC-6 cells in the absence or presence of Cr

**Figure S10**. Immunofluorescence detecting the expressions of claudin-1 and occludin in mice ileum

**Figure S11**. Bacterial counts in mice peripheral blood

**Figure S12**. Clustering heat map showing the alteration of proteins in irradiated IEC-6 cells after treatment with Cr

**Figure S13**. Viability of irradiated IEC-6 and HIEC-6 cells in the absence or presence of Fer-1

**Figure S14**. Viability of HIEC-6 cells treated with 5 µM erastin in the absence or presence of Cr or CC

**Figure S15**. Gray analysis showing the ACSL4 and COX2 contents in mice ileum

**Figure S16**. HE staining revealing the impact of RGX-202 administration on the radioprotection of DTRF

**Figure S17**. Cr contributes to the intestinal radioprotection of *B. pseudolongum*

**Table S1**. Relative abundance of top 10 bacterial species at the phylum level

| Taxonomy | ALF (%) | DTRF (%) |
| --- | --- | --- |
| Bacteroidota | 37.8656 | 55.3714 |
| Firmicutes | 47.0958 | 29.5171 |
| Verrucomicrobiota | 10.5226 | 9.0811 |
| Campilobacterota | 2.2673 | 2.3092 |
| Patescibacteria | 1.0977 | 0.3757 |
| Actinobacteriota | 0.4441 | 1.6738 |
| Proteobacteria | 0.197 | 0.8815 |
| Desulfobacterota | 0.1757 | 0.6424 |
| Deferribacterota | 0.0298 | 0.1017 |
| Spirochaetota | 0.2237 | 0.0389 |
| Others | 0.0887 | 0.0073 |

**Table S2**. Significantly changed bacteria in mice subjected to DTRF compared with that in mice subjected to ALF

| Species | *p* value^*^ | Change |
| --- | --- | --- |
| Erysipelotrichaceae_bacterium | 0.003947752 | Up |
| Kazachstania_unispora | 0.003947752 | Down |
| Clostridium_sp_CAG_632 | 0.003947752 | Down |
| Bifidobacterium_pseudolongum | **0.003947752** | **Up** |
| Prevotella_sp_PCHR | 0.003947752 | Down |
| Duncaniella_dubosii | 0.003947752 | Up |
| Bacteroides_sp_CAG_927 | 0.003947752 | Up |
| Muribaculaceae_bacterium | 0.006485308 | Up |
| Allobaculum_stercoricanis | 0.01040562 | Up |
| Allobaculum_fili | 0.01040562 | Up |
| Faecalibaculum_rodentium | 0.01040562 | Up |
| Bifidobacterium_animalis | 0.01040562 | Up |
| Allobaculum_mucilyticum | 0.01040562 | Up |
| Caudoviricetes_sp | 0.016309172 | Down |
| bacterium_01xD8_71 | 0.024974679 | Down |
| Lactobacillus_taiwanensis | 0.024974679 | Up |
| Muribaculum_intestinale | 0.024974679 | Up |
| Alistipes_muris | 0.037372988 | Up |

^*^ *p* values were determined using the Kruskal-Wallis rank test.

**Table S3**. Top 8 pathways enriched by KEGG based on the metabolomics results obtained in the positive ion mode

| Map ID | Map Title | *p* value |
| --- | --- | --- |
| map00830 | Retinol metabolism | 0.107769424 |
| map00590 | Arachidonic acid metabolism | 0.21886283 |
| map05215 | Prostate cancer | 0.21886283 |
| map00780 | Biotin metabolism | 0.254204213 |
| map04960 | Aldosterone-regulated sodium reabsorption | 0.254204213 |
| map00500 | Starch and sucrose metabolism | 0.330827068 |
| map00564 | Glycerophospholipid metabolism | 0.330827068 |
| map00600 | Sphingolipid metabolism | 0.330827068 |

**Table S4**. The specific criteria for DAI scoring.

| Score^*^ | Weight loss ratio (%) | Score | Stool viscosity | | | Bleeding stool |
| --- | --- | --- | --- | --- | --- | --- |
| 0 | 0 | 0 | Normal | | Normal | |
| 1 | 1-5 |  |  | |  | |
| 2 | 5-10 | 2 | Loose and soft | | Positive occult blood | |
| 3 | 10-15 |  |  | |  | |
| 4 | > 15 | 4 | | Watery | Dominant bleeding | |

**^*^** The DAI value is obtained by adding the scores of these three indicators and dividing by 3.

**Table S5**. Detailed information for the antibodies used in Western blotting

| Antibody | Article No. | Production company | Country |
| --- | --- | --- | --- |
| Anti-COX2/Cyclooxygenase 2 | ab179800 | Abcam | USA |
| ACSL4 Rabbit mAb | A20414 | ABclonal | UK |
| AMPKα Antibody | #2532 | CST | USA |
| Phospho-AMPKα (Thr172)  (40H9) Rabbit mAb | #2535 | CST | USA |
| Phospho-Acetyl-CoA-Carboxylase(Ser79) Antibody | #3661 | CST | USA |
| Acetyl-CoA Carboxylase Antibody | #3662 | CST | USA |
| β-Actin | ab8226 | Abcam | USA |


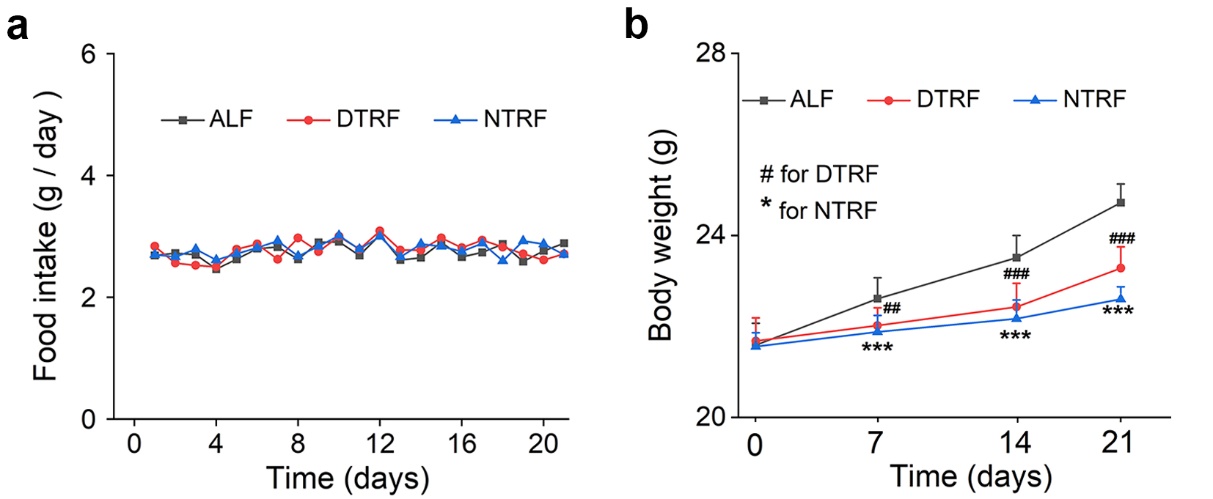


**Figure S1**. Food intake and body weight of mice subjected to ALF, DTRF or NTRF for 3 weeks. a) Food intake of mice subjected to ALF, DTRF, or NTRF. Each group contains ten mice. b) Body weight of mice before and after TRF. Each group contains ten mice. NTRF relative to ALF: ^***^, *p* < 0.001. DTRF relative to ALF: ^##^, *p* < 0.01; ^###^, *p* < 0.001.


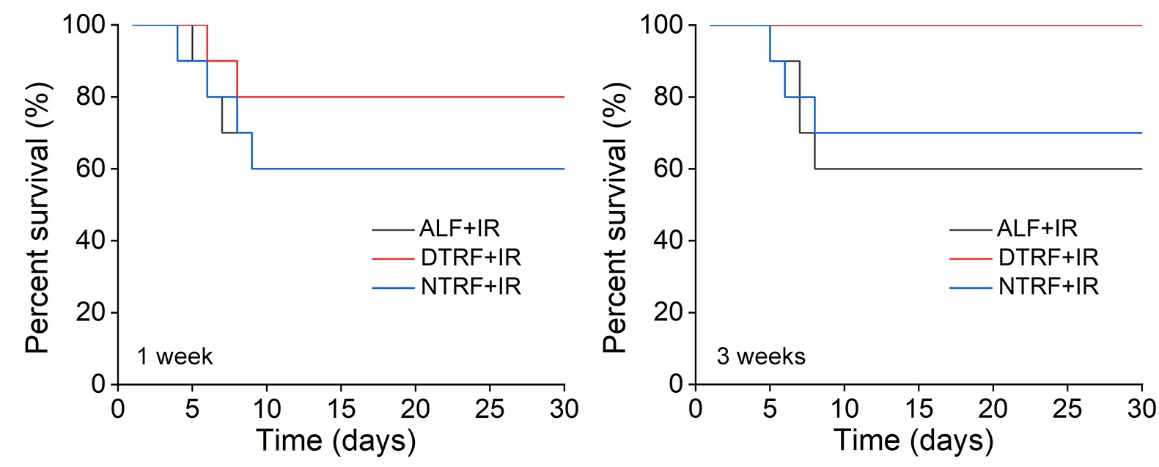


**Figure S2**. Percent survival of mice subjected to ALF, DTRF or NTRF for 1 and 3 weeks after TAI. Mouse survival was monitored for 30 days after irradiation. Each group contains 10 mice (n = 10).


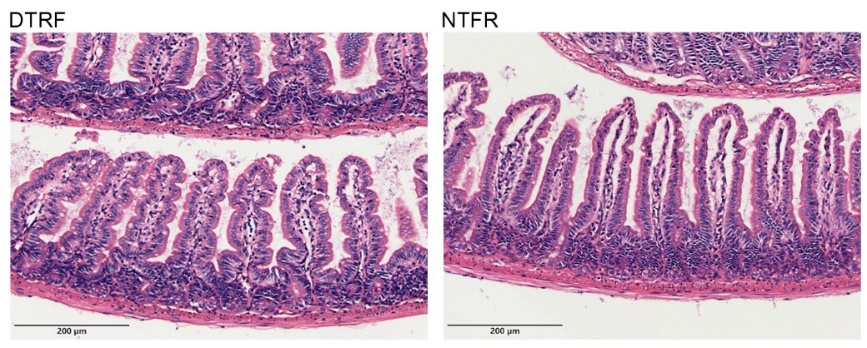


**Figure S3**. HE staining showing the ileum tissues of mice subjected to DTRF and NTRF. Scale indicates 200 μm.


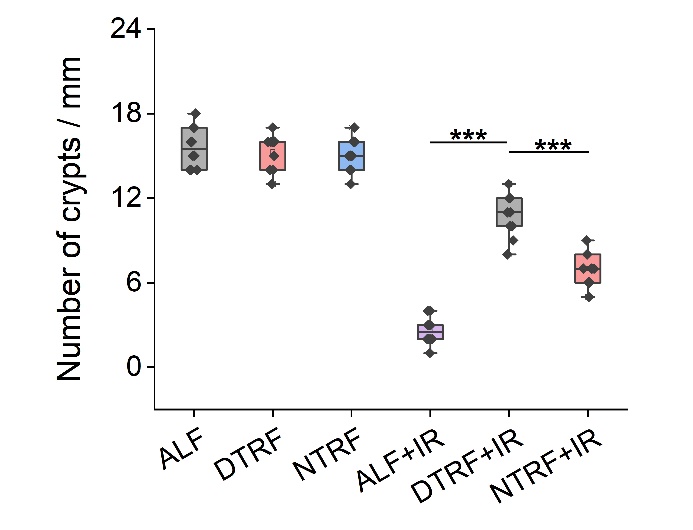


**Figure S4**. Number of crypts per millimeter in the ileum of mice subjected to ALF, DTRF, or NTRF. Ten fields of view were randomly selected for crypts counting. Results are shown in the boxplot. ^***^, *p* < 0.001.


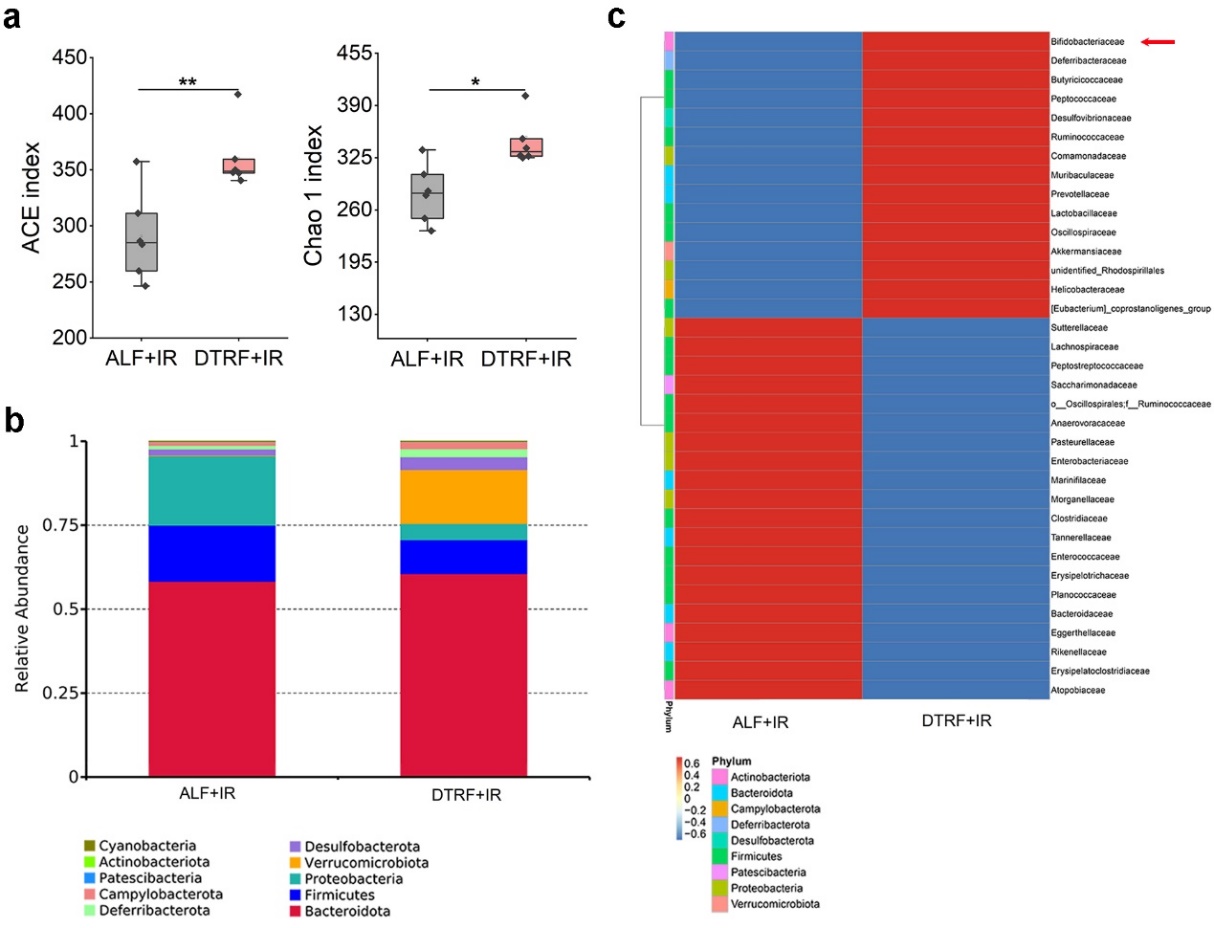


**Figure S5**. DTRF improves microbial diversity in irradiated mice. a) ACE and Chao 1 indices indicating the α diversity of mouse microbiota. ^*^, *p* < 0.05; ^**^, *p* < 0.01. b) Species abundance histogram showing the top 10 bacterial species at the phylum level. c) Clustering heat map showing the alteration of species after TAI and DTRF treatments. *Bifidobacteriaceae* is indicated by a red arrow.


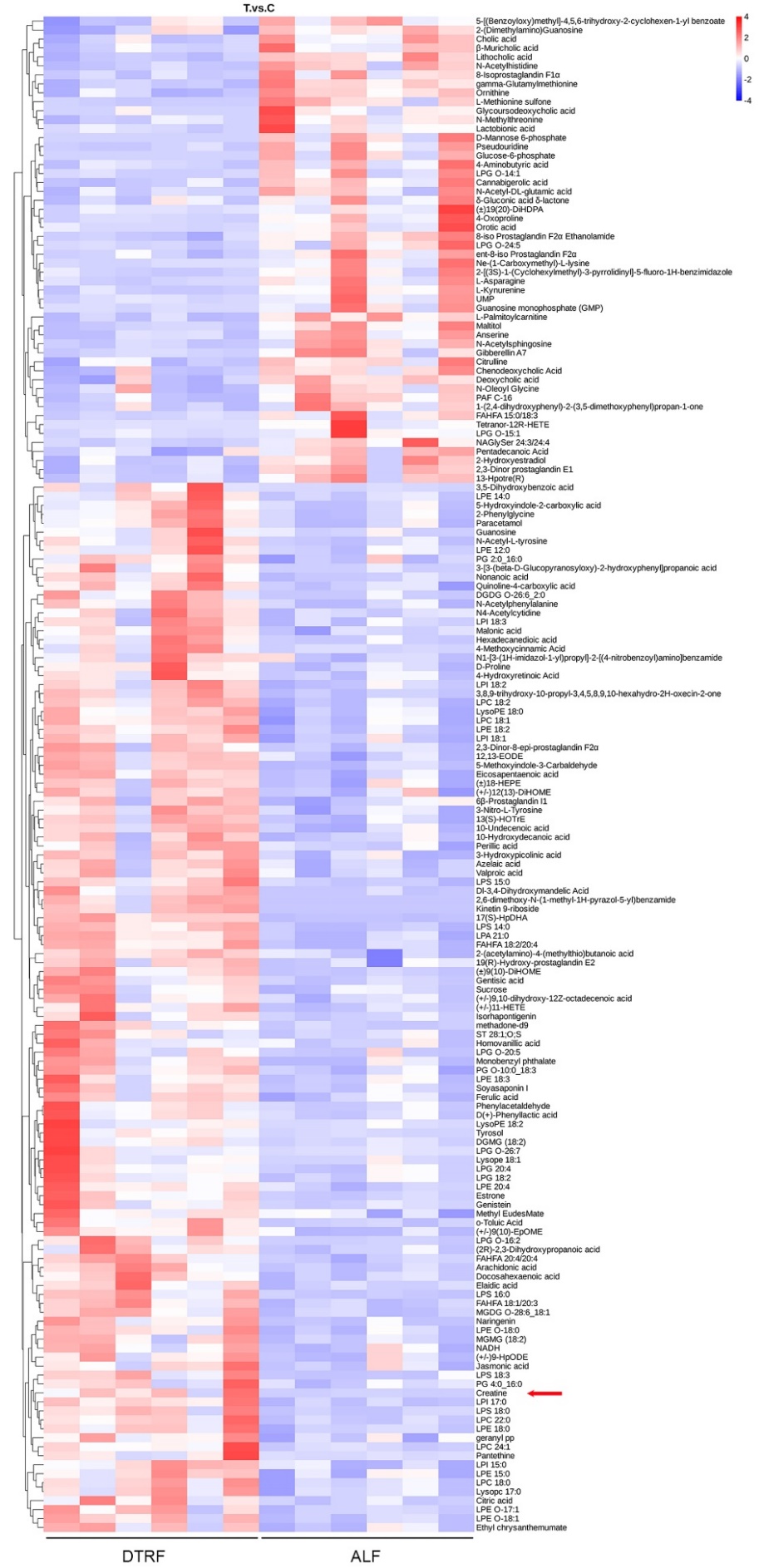


**Figure S6**. Clustering heat map showing the alteration of gut microbiota metabolites in mice after DTRF. Creatine is indicated by a red arrow.


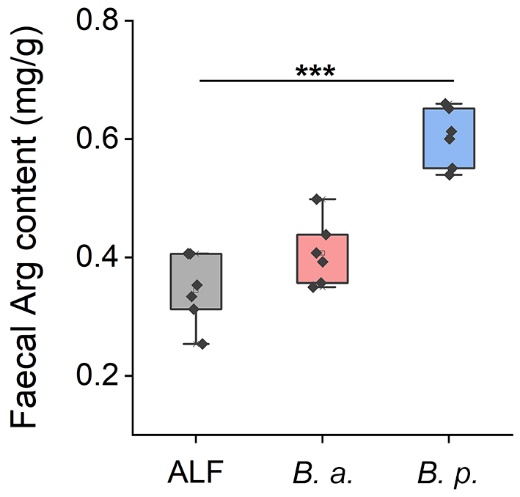


**Figure S7**. Faecal Arg contents in mice subjected to ALF after treatment with *B. animalis* or *B. pseudolongum. B.a., B. animalis*; *B.p.*, *B. pseudolongum*. Results are shown in the boxplot (n = 6). ^***^, *p* < 0.001


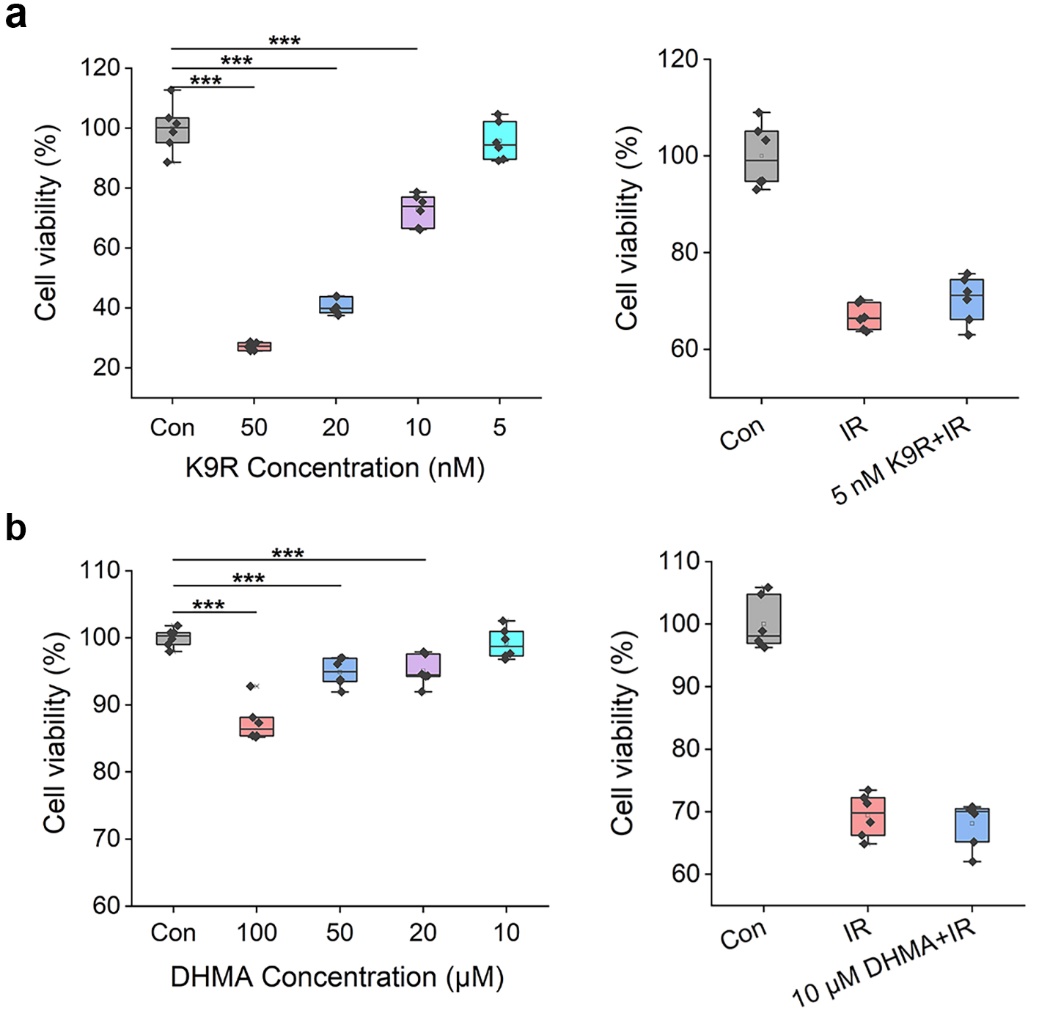


**Figure S8**. Influences of kinetin 9-riboside and dl-3,4-dihydroxymandelic acid on the survival of irradiated IEC-6 cells. a) Viability of IEC-6 cells exposed to kinetin 9-riboside in the absence and presence of IR. A significant cytotoxicity was detected for Kinetin 9-riboside (K9R) at concentrations above 5 nM. Results are shown in the boxplot (n = 6). ^***^, *p* < 0.001. K9R had no therapeutic effect on irradiated cells at 5 nM. b) Viability of IEC-6 cells exposed to dl-3,4-dihydroxymandelic acid (DHMA) in the absence and presence of IR. DHMA also had a significant cytotoxicity at concentrations above 10 µM. Results are shown in the boxplot (n = 6). ^***^, *p* < 0.001. Meanwhile, no therapeutic effect was detected for DHMA at 10 µM.


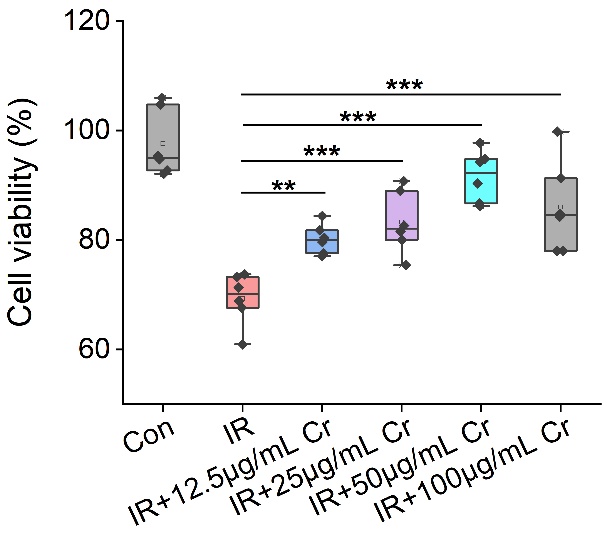


**Figure S9**. Viability of irradiated HIEC-6 cells in the absence or presence of Cr. Results are shown in the boxplot (n = 6). ^**^, *p* < 0.01; ^***^ *p* < 0.001.


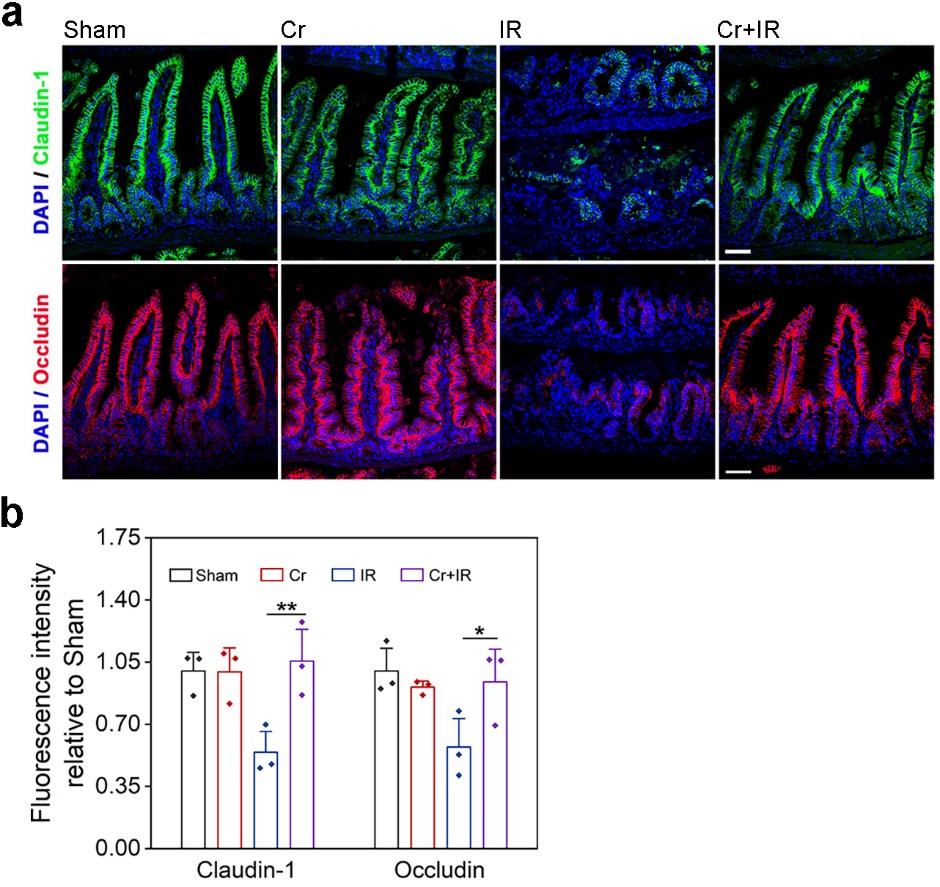


**Figure S10**. Immunofluorescence detecting the expressions of claudin-1 and occludin in mice ileum. a) Claudin-1 and occludin are traced with green and red fluorescence, respectively. Nucleus is stained by DAPI. The scale bar indicates 20 µm. b) Fluorescence intensity relative to the sham group is shown in the histogram. Results are presented as the means + SDs. ^*^, *p* < 0.05; ^**^, *p* < 0.01.


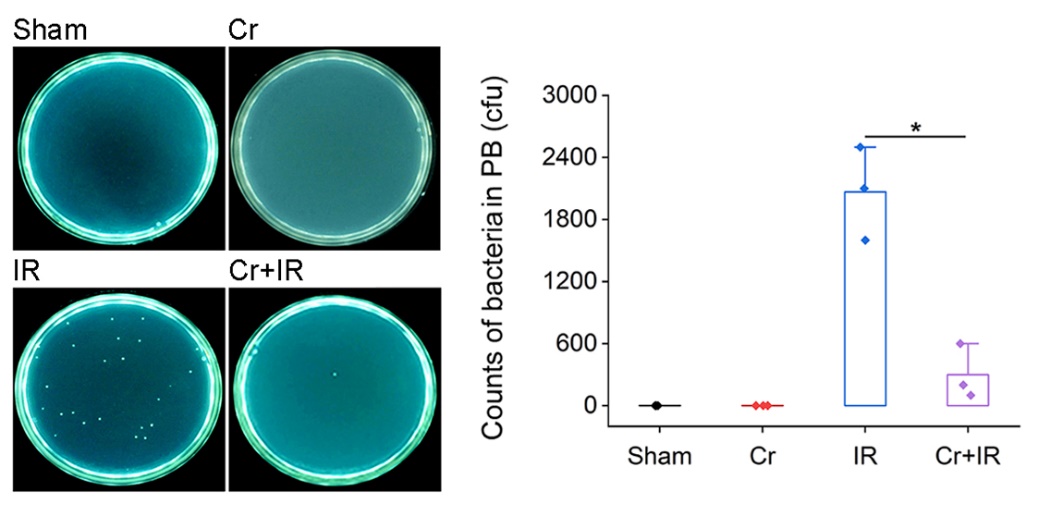


**Figure S11**. Bacterial counts in mice peripheral blood. Images are representative pictures showing the bacterial growth on culture plates. Statistical results are displayed in the histogram. ^*^, *p* < 0.05.


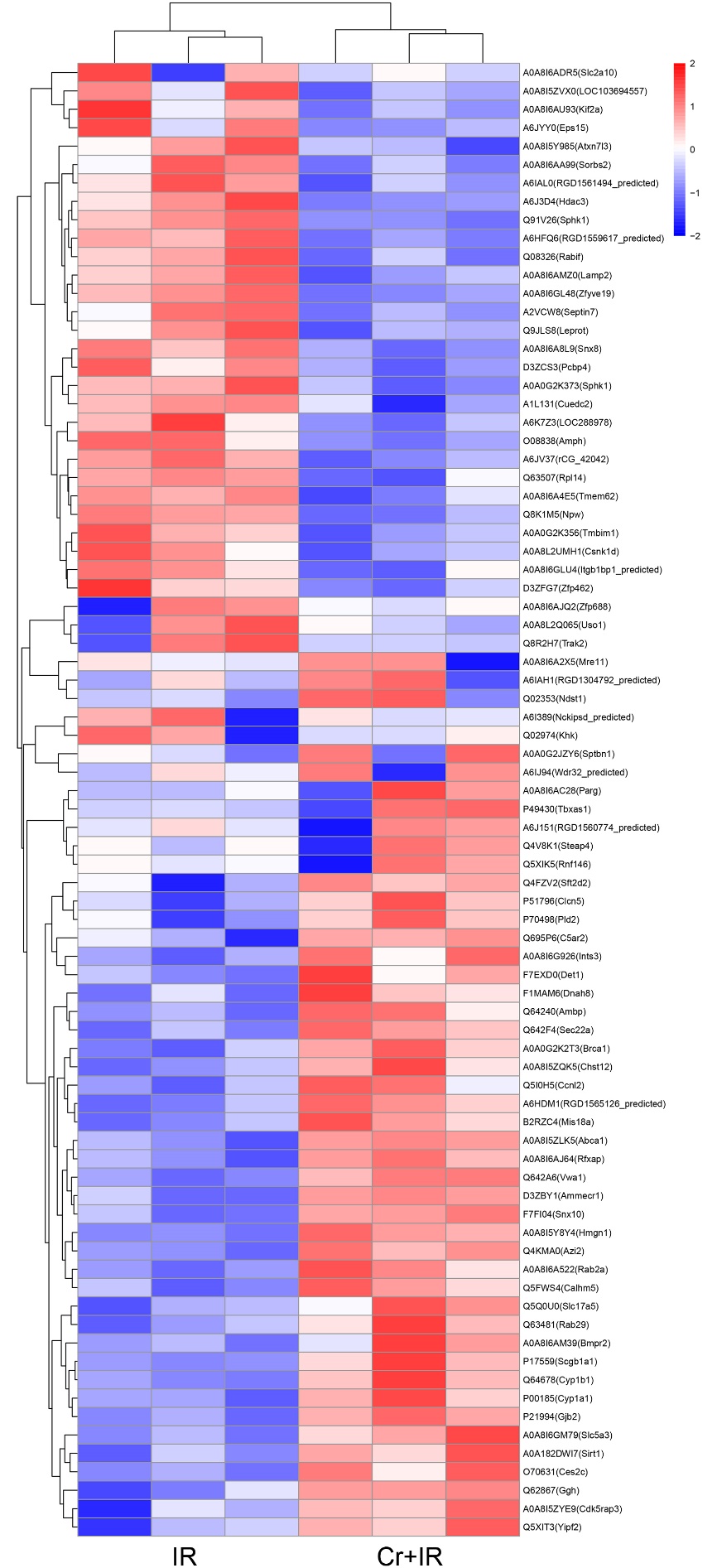


**Figure S12**. Clustering heat map showing the alteration of proteins in irradiated IEC-6 cells after treatment with Cr.


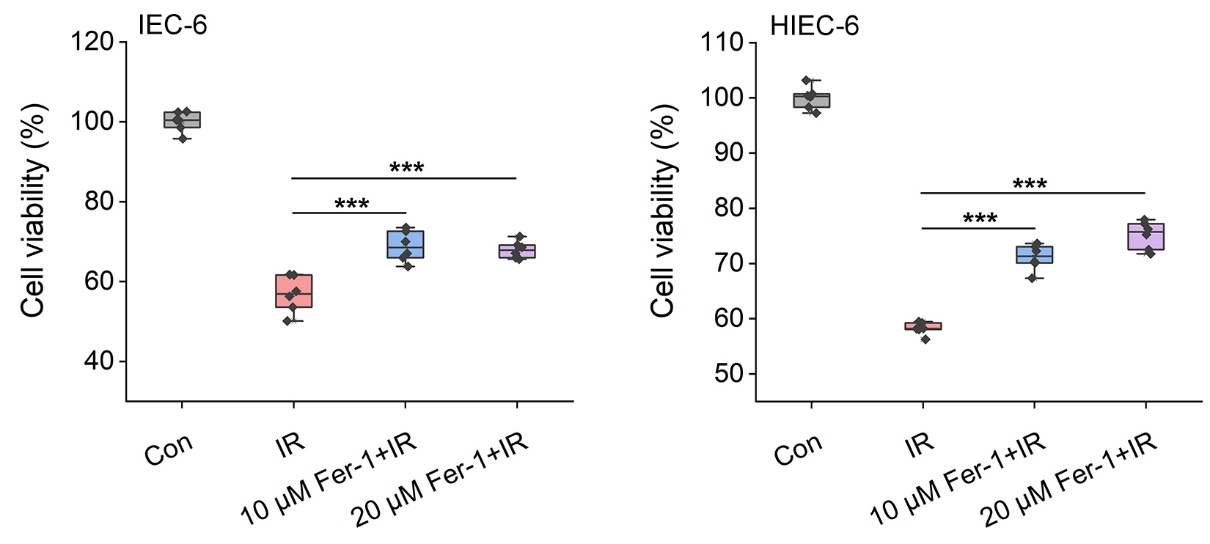


**Figure S13**. Viability of irradiated IEC-6 and HIEC-6 cells in the absence or presence of Fer-1. Results are shown in the boxplot (n = 6). ^***^, *p* < 0.001.


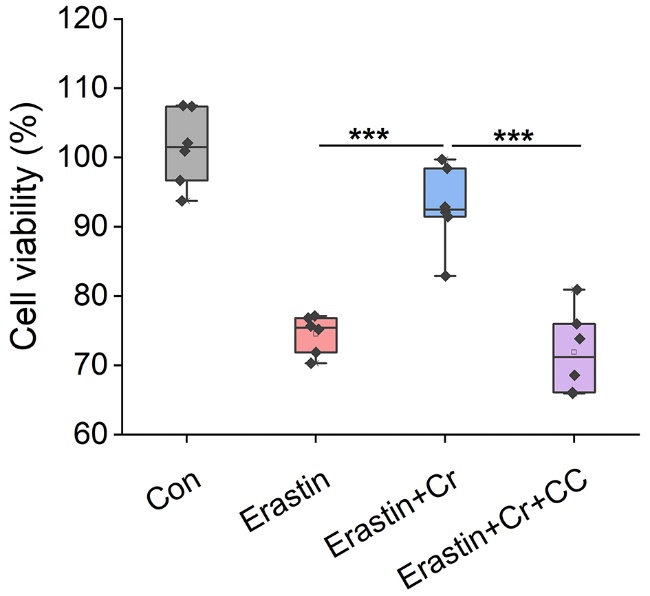


**Figure S14**. Viability of HIEC-6 cells treated with 5 µM erastin in the absence or presence of Cr or CC. The concentrations for Cr and CC were 25 µg/mL and 0.5 µM, respectively. Results are shown in the boxplot (n = 6). ^***^, *p* < 0.001.


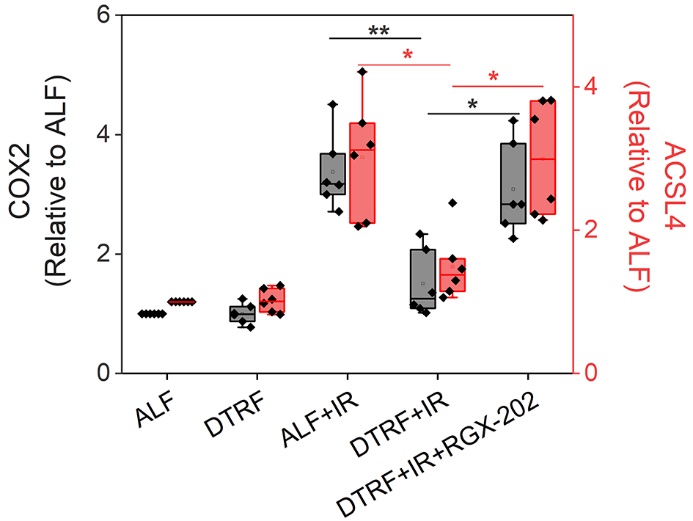


**Figure S15**. Gray analysis showing the ACSL4 and COX2 contents in mice ileum. β-Actin is the reference. Results are shown in the boxplot (n = 6). ^*^, *p* < 0.05; ^**^, *p* < 0.01.


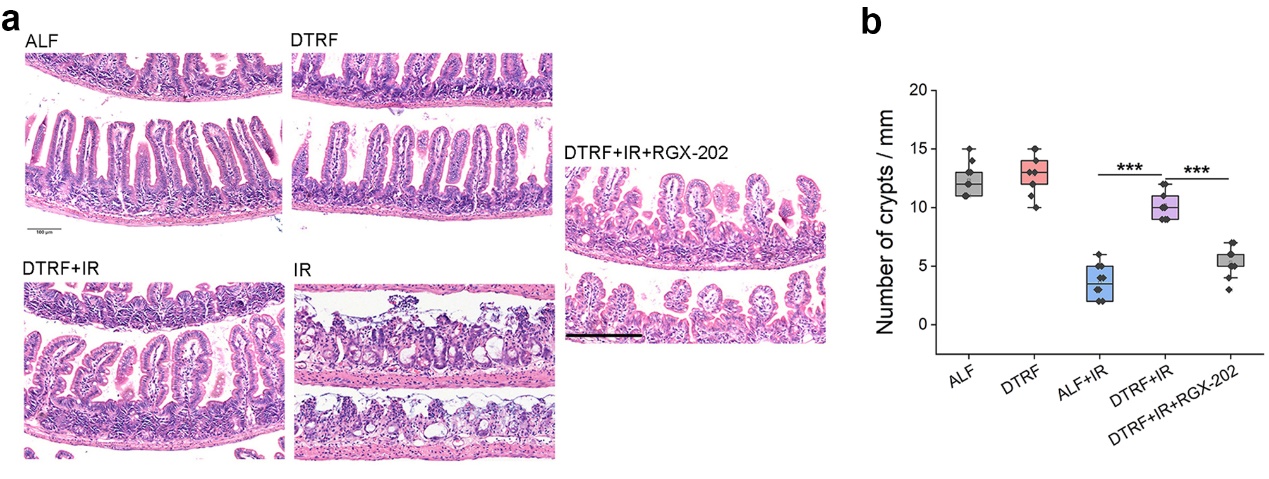


**Figure S16**. HE staining revealing the impact of RGX-202 administration on the radioprotection of DTRF. a) HE staining showing the ileum tissues of mice. Scale indicates 200 μm. b) Number of crypts per millimeter in mice ileum is shown in the boxplot. Ten fields of view were randomly selected for crypts counting. ^***^, *p* < 0.001.


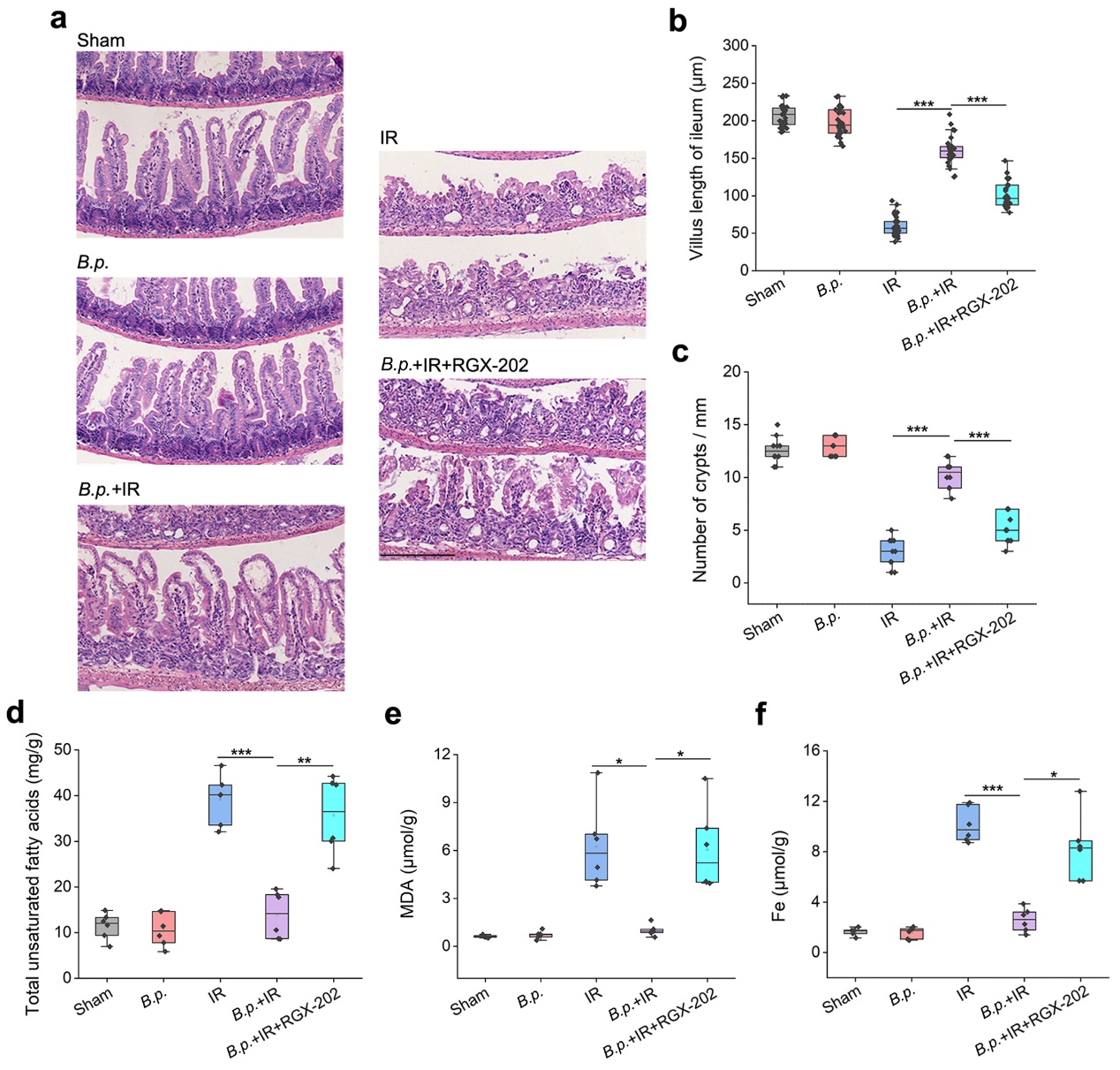


**Figure S17**. Cr contributes to the intestinal radioprotection of *B. pseudolongum*. a) HE staining showing the ileum tissues of mice. *B.p.*, *B. pseudolongum*. Scale indicates 200 μm. b) Measurement of the villous length in mice ileum. Results are shown in the boxplot (n = 50). ^***^, *p* < 0.001. c) Number of crypts per millimeter in mice ileum. Results are shown in the boxplot (n = 10). ^***^, *p* < 0.001. d) Total unsaturated fatty acids in mice ileum. Results are shown in the boxplot (n = 6). ^**^, *p* < 0.01; ^***^, *p* < 0.001. e) MDA contents in mice ileum. Results are shown in the boxplot (n = 6). ^*^, *p* < 0.05. f) Fe contents in mice ileum. Results are shown in the boxplot (n = 6). ^*^, *p* < 0.05; ^***^, *p* < 0.001.
